# Trends in characteristics and multi-product use among adolescents who use electronic cigarettes, United States 2011-2015

**DOI:** 10.1371/journal.pone.0177073

**Published:** 2017-05-05

**Authors:** Benjamin W. Chaffee, Elizabeth T. Couch, Stuart A. Gansky

**Affiliations:** Department of Preventive and Restorative Dental Sciences, Division of Oral Epidemiology and Dental Public Health, University of California San Francisco, San Francisco, California, United States of America; University College London, UNITED KINGDOM

## Abstract

**Objectives:**

Evaluate trends from 2011–2015 in electronic cigarette (e-cigarette) use among U.S. adolescents, grades 6–12, including prevalence and associations with past month use of cigarettes and other tobacco products, cigarette smoking intensity, quit attempts, and quit contemplation.

**Methods:**

Five consecutive waves from the National Youth Tobacco Survey (N = 101,011) were used to estimate the grade- and race/ethnicity-standardized prevalence of past month use of e-cigarettes and nine non e-cigarette tobacco products. We assessed linear trends by year and compared outcomes (e.g., tobacco use, smoking intensity) by e-cigarette past month use.

**Results:**

Past month e-cigarette use rose sharply from 2011–2015. In all years and both sexes, e-cigarette past month use and ever use were positively associated with use of cigarettes and other tobacco products, with past month e-cigarette use reaching 52% in 2015 among individuals who used ≥1 non e-cigarette tobacco product in the past month. Meanwhile, from 2011–2015, the population of adolescent past month e-cigarette users increasingly encompassed adolescents who were not past month users of other products (females: 19.0% to 41.7%; males: 11.1% to 36.7%) or had never used other products (females: 7.1% to 13.5%; males: 6.7% to 15.0%). Among male (but not female) past month cigarette users, there was a statistically significant positive association between past month e-cigarette use and daily cigarette smoking but not in all individual years. Past month e-cigarette use among past month cigarette smokers was not associated with cigarette quit attempts or quit contemplation, with no temporal trend.

**Conclusion:**

Adolescent past month e-cigarette use is associated with past month use of other tobacco but not with cigarette quit attempts or quit contemplation among cigarette users. Over five years, the average characteristics of U.S. adolescents who use e-cigarettes have shifted, increasingly including more adolescents who do not use non e-cigarette tobacco products.

## Introduction

Among the unknown public health implications of the expanding popularity of electronic cigarettes (e-cigarettes) are potential influences on adolescents’ use of conventional and emerging tobacco products, with possibilities ranging from displacement to catalysis of tobacco product use [1,2]. That e-cigarettes will reduce population-wide morbidity and mortality as a less harmful alternative to combustible tobacco is plausible but uncertain [3], and at least partly depends on the extent of e-cigarette uptake by adolescents who would not otherwise smoke cigarettes [4].

Over the past 5 years, adolescent e-cigarette ever and past month use has risen sharply [5–7]. Largely unregulated e-cigarette marketing and the extensive array of product flavors may disproportionately appeal to youth [8–11]. Concerns regarding adolescent e-cigarette use include exposure to potentially harmful toxins, renormalization of smoking behavior, nicotine dependence, and subsequent initiation and long-term use of cigarettes and other tobacco products [10–12]. Effective August 2016, the Food and Drug Administration finalized a rule deeming the agency's authority to regulate e-cigarettes and other tobacco products in the United States [13].

Despite major increases in e-cigarette ever and past month use, few investigations have examined how the behaviors and characteristics of adolescent e-cigarette users have evolved over time, including with regard to adolescent e-cigarette use and concurrent use of tobacco. Cross-sectionally, multiple large surveys have shown that adolescents who use e-cigarettes are more likely to use combustible tobacco products than e-cigarette non-users [14–18]. Poly-use of e-cigarettes and multiple tobacco products within the previous 30 days involves potentially complex patterns in use frequency and product combinations [18]: patterns that may be evolving over time.

In addition, relatively little is known regarding e-cigarette use and conventional cigarette smoking intensity or quitting behavior among youth. In Europe, among adolescent cigarette users, past month e-cigarette use was associated with more frequent smoking [19]. In large national surveys conducted in 2011 and 2012, ever and past month e-cigarette use was positively associated with cigarette smoking and negatively associated with smoking abstinence among cigarette ever-users in the U.S. [20] and Republic of Korea [21].

In the present study, we examined secular trends related to e-cigarette ever and past month use over five annual National Youth Tobacco Survey (NYTS) waves from 2011–2015. Such analysis covers a time period during which e-cigarette use grew increasingly prevalent among adolescents. Recent tendencies might help to predict future characteristics of the adolescent e-cigarette using population. Any changes in the relationships with use of tobacco products, particularly cigarettes, could provide insight as to how e-cigarette emergence might influence adolescents’ other tobacco-related behaviors.

Our research objectives were to evaluate trends from 2011–2015 in e-cigarette ever and past month use among U.S. adolescents, grades 6–12, including: 1) prevalence of e-cigarette ever use and past 30-day use; 2) the relationships between past 30-day use of both e-cigarettes and other tobacco products; and 3) among cigarette users, the associations between past 30-day e-cigarette use and cigarette smoking intensity, quit attempts, and quit intentions.

## Methods

The present study analyzed repeated cross-sectional survey data from the National Youth Tobacco Survey (NYTS). The U.S. Centers for Disease Control and Prevention and the U.S. Food and Drug Administration administer the NYTS to monitor selected tobacco indicators used in informing comprehensive tobacco prevention and control programs. The NYTS is a nationally representative, school-based survey, which began in 1999, of private and public U.S. middle school (grades 6–8) and high school (grades 9–12) students. The NYTS uses a stratified, three-stage cluster sampling design for a voluntary self-administered survey with parental permission. Detailed survey methodology is available elsewhere [22]. The present analysis included five annual survey waves beginning in 2011, the first year that the NYTS featured items for e-cigarettes. Study reporting followed STROBE guidelines [23]. De-identified study data are freely available on the Internet [22]. The present analysis included no participant contact and generated no identifiable health information.

### Study variables

The 2011–2015 waves of the NYTS include questionnaire items for ever use (lifetime, even once) and past 30-day use (≥1 day in the previous month) for e-cigarettes in addition to nine tobacco products: cigarettes, cigars (including little cigars and cigarillos), conventional smokeless tobacco (chewing tobacco and oral snuff), tobacco pipes, bidis, kreteks (clove cigars; not included in 2014 or 2015), snus, dissolvable tobacco, and hookah (tobacco waterpipe). In 2011–2013, e-cigarette ever use was determined by choosing the option “Electronic Cigarettes or E-cigarettes, such as Ruyan or NJOY” following the question, “Which of the following tobacco products have you ever tried, even just one time?” and past 30-day use determined by choosing that same option after the question, “During the past 30 days, which of the following tobacco products did you use on at least one day?” In 2014, e-cigarette ever use was determined from the survey item, “Have you ever tried an electronic cigarette or e-cigarette such as blu, 21st Century Smoke or NJOY?” and past 30-day use from the item, “During the past 30 days, on how many days did you use electronic cigarettes or e-cigarettes such as blu, 21st Century Smoke, or NJOY?” with seven categorical response options from 0 days to all 30 days, matching the question format used for tobacco products such as cigarettes and smokeless tobacco. In 2015, e-cigarette use questions were in the same format as 2014 but were preceded by a brief written description of how e-cigarettes may look, how they may be used, alternative names, and example brands. In all five years, use of other tobacco products, such as hookah, snus, and dissolvable tobacco, was determined from the same item that was used in 2011–2013 for e-cigarettes, although the number and order of listed responses differed in some waves. We considered two products to be used concurrently if each was used at least one day in the previous 30 days.

Among past 30-day cigarette users, the intensity of cigarette smoking was assessed according to the number of days that participants smoked in the past month (all 30 days or <30 days) and the number of cigarettes smoked on days that cigarettes were used (>10 per day or ≤10). All participants were asked how many times they had tried to quit cigarettes for at least one day in the past twelve months (≥1 time was considered a quit attempt). In separate items, all were asked whether they “are seriously thinking about quitting cigarettes” or “seriously thinking about quitting the use of all tobacco products.” Answers were dichotomized as yes or no, regardless of whether participants indicated that they planned to quit within 30 days, 6 months, 12 months, or later.

### Study size

The NYTS sample size is designed to support estimates for tobacco-related knowledge, attitudes, and behaviors with precision of ±5% for 95% confidence intervals (CIs), including within subgroups defined by sex, grade, and race/ethnicity [22]. We pooled data over all grades (6–12) due to small numbers of e-cigarette users prior to 2014. As a sensitivity check, we repeated all analyses separately for the middle school and high school samples, and trends were not substantially different; however, tobacco use was consistently more prevalent among high school students.

Analyses were restricted to the 50,034 female and 50,977 male participants in the five NYTS waves from 2011–2015 (total N = 101,011). Across the ten products assessed in the NYTS, data were missing for 2.9% of all use questions, affecting 11.4% of participants (8.5% for ever use and 7.6% for past 30-day use). To avoid excluding these individuals in deriving variables such as never use of any product (as would occur in complete-case analysis), we multiply imputed missing values by chained equations, including missing grade in school (0.3% of participants) and race/ethnicity (3.8%), averaging all point estimates across 10 imputations and correcting variances with the standard formula [24].

### Statistical methods

Analyses were conducted separately for males and females. To account for sampling design, all analyses used NYTS standardized survey weights and Taylor series-linearized variance estimation in Stata 14 (StataCorp LP, College Station, USA). Model-based standardized marginal prevalences [25] were obtained by year, using survey logistic regression models (command: svy linearized) fitted with survey weights for the outcome of interest (e.g., past month cigarette use) with indicator variables for year, grade, and race/ethnicity (Black/African American, Hispanic/Latino, White, or other). Linear trends were assessed from survey logistic regression models with a continuous term for year. Additionally, standardized marginal prevalences of outcome variables (e.g., cigarette quit attempt) were tested by comparison group (e.g., past month e-cigarette use) using the Chi-squared test, with standard errors adjusted for multiple imputation. Given the large number of hypothesis tests, we set a conservative threshold of P<0.001 to indicate statistical significance.

### Sensitivity checks

Due to changes in e-cigarette question formatting after 2013, we repeated the trends analysis restricted to years 2011–2013. Additionally, complete-case analyses were conducted, in which participants with missing data for grade, race/ethnicity, or tobacco use variables were excluded, as opposed to imputing missing values.

## Results

### Overall trends in ever and past month e-cigarette and other tobacco use

**Fig 1:**
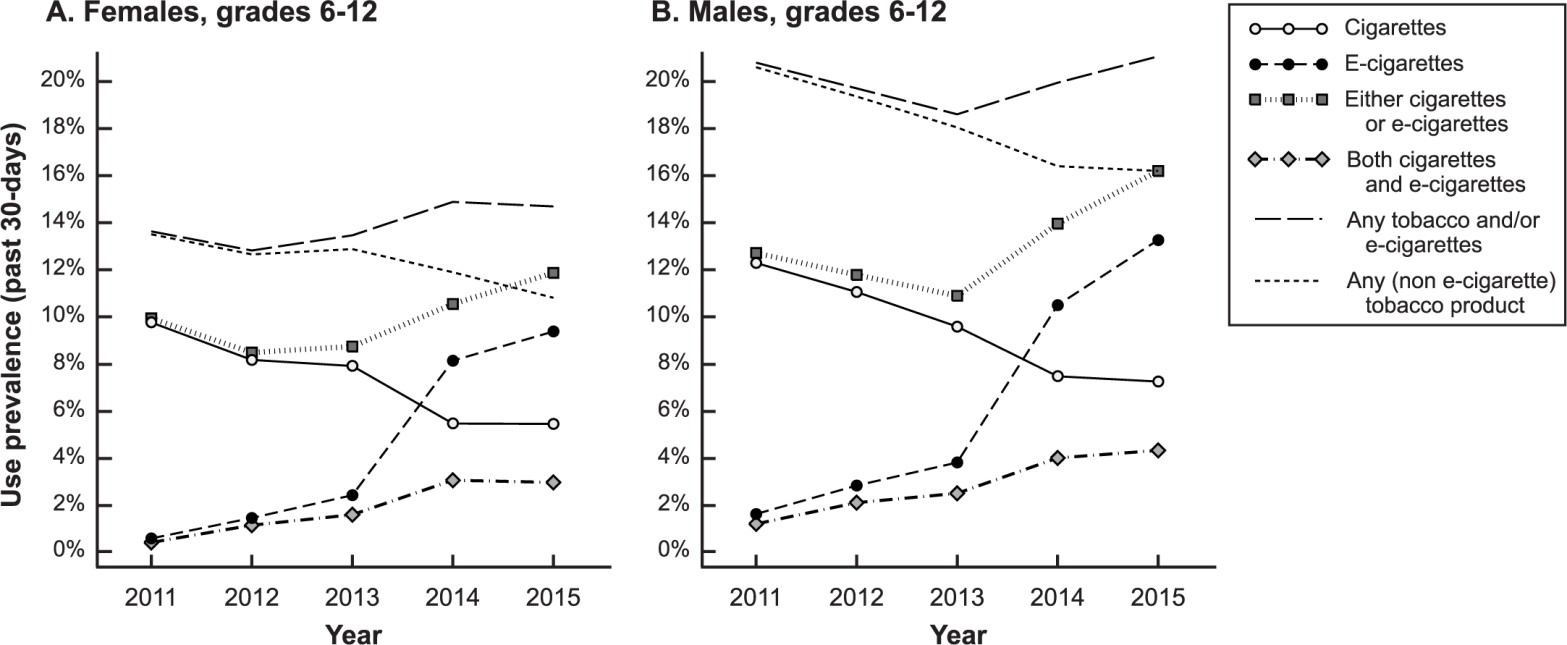
Prevalence of past 30-day cigarette use, past 30-day e-cigarette use, and past 30-day use of other products in the National Youth Tobacco Survey, 2011–2015. Prevalence of past month use of cigarettes and e-cigarettes are shown for (A) females, N = 50,034 and (B) males, N = 50,977. As a result of increasing e-cigarette use, the percentage of individuals using either product increased, even as cigarette use declined. “Either” refers to use of at least one cigarette or e-cigarette on ≥1 day of the previous 30 days. “Both” refers to use of cigarettes ≥1 day of the previous 30 days and use of e-cigarettes on ≥1 day of the previous 30 days, not necessarily the same day. "Any" tobacco and/or e-cigarettes refers to use of ≥1 of cigarettes, e-cigarettes, cigars (including little cigars and cigarillos), conventional smokeless tobacco (chewing tobacco and oral snuff), tobacco pipes, bidis, kreteks (not included in 2014 or 2015), snus, dissolvable tobacco, or hookah on ≥1 day of the previous 30 days, not necessarily the same day. "Any (non e-cigarette)" product excludes e-cigarettes from the previous category. Marginal percentages adjusted across years for grade in school and race/ethnicity.

From 2011–2015, past 30-day cigarette use declined among both females and males, but past 30-day use of e-cigarettes increased dramatically (Fig 1). In 2014 and 2015, past 30-day e-cigarette use exceeded past 30-day cigarette use (Fig 1, in 2015: 9.4% e-cigarettes versus 5.4% cigarettes for females; 13.2% e-cigarettes versus 7.2% cigarettes for males). The percentage of the grade 6–12 population that used either cigarettes or e-cigarettes at least once in the prior month was greater in 2014 and 2015 than in any of the three years prior. The prevalence of using both cigarettes and e-cigarettes at least one day in the past month (not necessarily the same day) rose 7.3-fold among females (from 0.4% to 3.0%) and 3.7-fold among males (from 1.2% to 4.3%). Among past 30-day cigarette users, past month e-cigarette use rose sharply from 2011 to 2015: from 4.3% to 53.6% among females, and from 9.7% to 59.5% among males (not shown in tables). Past month use of ≥1 non e-cigarette tobacco product was lower in 2015 than in 2011 for both females and males but not statistically significantly different from 2013 or 2014 (Fig 1).

Overall, e-cigarette ever use increased 10-fold among females (from 2.5% in 2011 to 24.8% in 2015) and nearly 7-fold among males (from 4.3% in 2011 to 29.4% in 2015) (Table 1). However, the percentage of past 30-day e-cigarette users that were male declined (not shown in tables): in 2011, nearly three-quarters of past month e-cigarette users were male (74.7%), but by 2015, past month e-cigarette users were more evenly divided by sex (59.8% male). Past month e-cigarette use increased in all racial/ethnic groups (S1 File). For females and males, past 30-day e-cigarette use was more prevalent among individuals who identified as Hispanic/Latino or White than among those identifying as Black or other (S1 File).

**Table 1:** Electronic cigarette ever use and past month use according to ever use (yes or no) and past month use (yes or no) of other tobacco products, 2011–2015.

	**Females, grades 6–12**											
	Overall (n: sample size)		Ever use of any other tobacco^1^				p-value^2^	Past 30-day use of any other tobacco^1^				p-value^2^
			Yes (n: sample size)		No (n: sample size)			Yes (n: sample size)		No (n: sample size)		
***Ever use of e-cigarettes*:**												
	n	% e-cigarette ever (SE)	n	% e-cigarette ever (SE)	n	% e-cigarette ever (SE)		n	% e-cigarette ever (SE)	n	% e-cigarette ever (SE)	
All years	50,034	11.6 (0.3)	15,226	31.9 (0.6)	34,808	2.8 (0.2)	<0.001	6,213	41.7 (1.0)	43,821	7.4 (0.3)	<0.001
2011	9,315	2.5 (0.3)	3,091	7.2 (0.7)	6,224	0.2 (0.1)	<0.001	1,263	12.5 (1.4)	8,052	0.9 (0.2)	<0.001
2012	12,275	5.6 (0.5)	3,622	17.5 (1.2)	8,653	0.5 (0.1)	<0.001	1,510	27.1 (1.8)	10,765	2.5 (0.3)	<0.001
2013	9,177	7.0 (0.5)	2,931	21.7 (1.4)	6,246	0.7 (0.2)	<0.001	1,230	32.9 (2.3)	7,947	3.3 (0.4)	<0.001
2014	10,645	18.1 (1.0)	3,113	50.7 (2.0)	7,532	4.4 (0.6)	<0.001	1,272	66.1 (2.6)	9,373	11.6 (0.9)	<0.001
2015	8,622	24.8 (1.0)	2,469	65.8 (1.6)	6,153	7.9 (0.6)	<0.001	938	78.9 (2.5)	7,684	18.3 (0.9)	<0.001
Trend^3^		<0.001		<0.001		<0.001			<0.001		<0.001	
***Past month use of e-cigarettes*:**												
	n	% e-cig. past month (SE)	n	% e-cig. past month (SE)	n	% e-cig. past month (SE)		n	% e-cig. past month (SE)	n	% e-cig. past month (SE)	
All years	50,034	4.4 (0.2)	15,226	12.7 (0.5)	34,808	0.8 (0.1)	<0.001	6,213	23.0 (0.9)	43,821	1.8 (0.1)	<0.001
2011	9,315	0.6 (0.1)	3,091	1.6 (0.3)	6,224	0.1 (0.0)	<0.001	1,263	3.4 (0.7)	8,052	0.1 (0.1)	<0.001
2012	12,275	1.5 (0.2)	3,622	4.6 (0.5)	8,653	0.1 (0.1)	<0.001	1,510	10.4 (1.1)	10,765	0.2 (0.1)	<0.001
2013	9,177	2.4 (0.3)	2,931	7.4 (0.8)	6,246	0.3 (0.2)	<0.001	1,230	14.5 (1.6)	7,947	0.7 (0.2)	<0.001
2014	10,645	8.1 (0.7)	3,113	23.6 (1.9)	7,532	1.5 (0.4)	<0.001	1,272	42.7 (2.7)	9,373	3.4 (0.5)	<0.001
2015	8,622	9.4 (0.6)	2,469	27.6 (1.5)	6,153	1.8 (0.3)	<0.001	938	51.5 (3.0)	7,684	4.3 (0.4)	<0.001
Trend^3^		<0.001		<0.001		<0.001			<0.001		<0.001	
	**Males, grades 6–12**											
	Overall (n: sample size)		Ever use of any other tobacco^1^				p-value^2^	Past 30-day use of any other tobacco^1^				p-value^2^
			Yes (n: sample size)		No (n: sample size)			Yes (n: sample size)		No (n: sample size)		
***Ever use of e-cigarettes*:**												
	n	% e-cigarette ever (SE)	n	% e-cigarette ever (SE)	n	% e-cigarette ever (SE)		n	% e-cigarette ever (SE)	n	% e-cigarette ever (SE)	
All years	50,977	14.6 (0.4)	18,574	34.4 (0.6)	32,403	3.4 (0.2)	<0.001	9,219	43.8 (1.0)	41,758	8.1 (0.3)	<0.001
2011	9,284	4.3 (0.5)	3,793	10.2 (1.1)	5,491	0.5 (0.2)	<0.001	1,897	16.9 (1.8)	7,387	1.0 (0.2)	<0.001
2012	12,369	8.3 (0.6)	4,566	21.7 (1.4)	7,803	0.4 (0.1)	<0.001	2,363	32.4 (2.2)	10,006	2.6 (0.3)	<0.001
2013	9,216	9.5 (0.6)	3,438	24.7 (1.3)	5,778	0.7 (0.2)	<0.001	1,690	37.6 (1.8)	7,526	3.3 (0.3)	<0.001
2014	11,150	21.5 (1.0)	3,822	52.5 (1.9)	7,328	5.3 (0.6)	<0.001	1,826	63.9 (2.8)	9,324	13.0 (0.8)	<0.001
2015	8,958	29.4 (1.1)	2,955	69.5 (1.7)	6,003	9.5 (0.7)	<0.001	1,443	78.2 (2.9)	7,515	19.8 (0.9)	<0.001
Trend^3^		<0.001		<0.001		<0.001			<0.001		<0.001	
***Past month use of e-cigarettes*:**												
	n	% e-cig. past month (SE)	n	% e-cig. past month (SE)	n	% e-cig. past month (SE)		n	% e-cig. past month (SE)	n	% e-cig. past month (SE)	
All years	50,977	6.4 (0.3)	18,574	15.7 (0.6)	32,403	1.1 (0.1)	<0.001	9,219	24.8 (0.9)	41,758	2.3 (0.2)	<0.001
2011	9,284	1.6 (0.3)	3,793	3.8 (0.6)	5,491	0.2 (0.1)	<0.001	1,897	6.9 (1.1)	7,387	0.2 (0.1)	<0.001
2012	12,369	2.8 (0.3)	4,566	7.2 (0.8)	7,803	0.2 (0.1)	<0.001	2,363	12.7 (1.3)	10,006	0.5 (0.1)	<0.001
2013	9,216	3.8 (0.4)	3,438	9.6 (1.0)	5,778	0.3 (0.1)	<0.001	1,690	17.9 (1.8)	7,526	0.7 (0.2)	<0.001
2014	11,150	10.4 (0.8)	3,822	26.9 (2.1)	7,328	1.9 (0.3)	<0.001	1,826	42.0 (3.2)	9,324	4.2 (0.5)	<0.001
2015	8,958	13.2 (0.8)	2,955	34.1 (1.9)	6,003	2.9 (0.4)	<0.001	1,443	51.9 (3.0)	7,515	5.7 (0.6)	<0.001
Trend^3^		<0.001		<0.001		<0.001			<0.001		<0.001	

In all years, e-cigarette use (ever and past 30-days) was associated with use of other tobacco products, but e-cigarette use prevalence increased from 2011–2015 among both other tobacco users and other tobacco non-users.

1. Other tobacco includes: cigarettes, cigars, pipes, bidis, kreteks (2011–2013), conventional smokeless tobacco, snus, dissolvable tobacco, and hookah.

2. Chi-square test for difference in use of e-cigarettes between users and non-users of other tobacco products

3. P-for-linear trend, 2011–2015

Marginal percentages adjusted across years for grade in school and race/ethnicity.

Subgroup sample sizes (n) are the mean sample size over 10 imputations for missing value assignment.

Abbreviations: e-cig. = e-cigarette; n = sample size (denominator); SE = standard error

### Trends in ever and past month e-cigarette use according to ever and past month use of other tobacco products

Ever and past month e-cigarette use was strongly associated with ever and past month use of cigarettes and other tobacco products (Table 1). In addition to this association, e-cigarette ever and past month use increased significantly from 2011–2015 among both other tobacco users and other tobacco non-users in both sexes (all trends P<0.001) (Table 1). In 2015, approximately two-thirds of adolescents who had ever tried any other tobacco product had also tried e-cigarettes. Among past 30-day users of at least one other tobacco product, the percentage of past 30-day e-cigarette users increased significantly from 2011–2015: from 3.4% to 51.5% among females, and from 6.9% to 51.9% among males (Table 1).

In 2015, no tobacco product had higher past 30-day use prevalence than e-cigarettes among individuals who had never tried any other product. Among individuals who had never used any other product, prevalence of past 30-day e-cigarette use increased by an order of magnitude from 2011–2015: 0.1% to 1.8% for females; 0.2% to 2.9% for males (Table 1). In 2015, for females, no other product exceeded 0.5% prevalence of past month use among never-users of anything else; for males, only past month use of conventional smokeless tobacco (0.6%) or cigars (0.6%) was above 0.5% among never-users of anything else (not shown in tables). Among past 30-day e-cigarette users, the percentage that had never used any other product increased from 2011–2015 among females (7.1% to 13.5%; P-for-linear-trend: 0.024) and males (6.7% to 15.0%; P-for-linear-trend: <0.001) (not shown in tables).

### Trends in past month use of other tobacco products according to past month e-cigarette use

In all years and both sexes, past 30-day use of other tobacco products, including cigarettes, non-cigarette combustibles, smokeless tobacco, and hookah, was significantly more common among past month e-cigarette users than past month non-users (Table 2). From 2011–2015, past month e-cigarette users were decreasingly likely also to be past month users of other tobacco products, with the exception of hookah. Use of combustible and smokeless tobacco products also declined significantly among past month e-cigarette non-users (Table 2). The percentage of past month e-cigarette users who were also past month users of any other tobacco product declined from 81.0% (females) and 88.9% (males) in 2011 to 58.3% (females) and 63.3% (males) in 2015 (Table 2). Importantly, the decreasing prevalence of multi-product use among past month e-cigarette users did not occur among a static denominator of e-cigarette users, but rather, coincided with expanding e-cigarette use by a larger portion of the total adolescent population. Thus, although the percentage of past month other tobacco use declined when comparing the subpopulations of the past month e-cigarette users from year to year (Table 2), the overall prevalence of using both e-cigarettes and other tobacco in the past month increased (Fig 1).

**Table 2:** Past month use of other tobacco products according to past month use (yes or no) of electronic cigarettes, 2011–2015.

	Females, grades 6–12							Males, grades 6–12						
	Overall (n: sample size)		Past 30-day e-cigarette use				^1^	Overall (n: sample size)		Past 30-day e-cigarette use				^1^
			Yes (n: sample size)		No (n: sample size)					Yes (n: sample size)		No (n: sample size)		
***Any other tobacco in past month*:**														
	n	% other tobacco (SE)	n	% other tobacco (SE)	n	% other tobacco (SE)		n	% other tobacco (SE)	n	% other tobacco (SE)	n	% other tobacco (SE)	
All years	50,034	12.2 (0.3)	2,094	64.5 (1.9)	47,940	9.8 (0.3)	*	50,977	18.0 (0.4)	3,183	70.2 (1.3)	47,794	14.5 (0.4)	*
2011	9,315	13.4 (0.8)	69	81.0 (7.6)	9,246	12.9 (0.7)	*	9,284	20.5 (1.0)	130	88.9 (4.1)	9,154	19.2 (0.9)	*
2012	12,275	12.6 (0.7)	180	89.0 (3.3)	12,095	11.4 (0.6)	*	12,369	19.3 (0.8)	342	87.6 (3.2)	12,027	17.2 (0.7)	*
2013	9,177	12.8 (0.6)	213	74.6 (5.1)	8,964	11.3 (0.6)	*	9,216	18.0 (1.0)	332	85.2 (2.7)	8,884	15.3 (0.9)	*
2014	10,645	11.9 (0.6)	849	63.0 (2.8)	9,796	7.5 (0.5)	*	11,150	16.4 (0.8)	1,183	66.2 (2.3)	9,967	10.8 (0.8)	*
2015	8,622	10.6 (0.8)	783	58.3 (3.5)	7,839	5.8 (0.6)	*	8,958	16.1 (1.0)	1,196	63.3 (2.1)	7,762	9.2 (1.0)	*
Trend^2^		0.008		<0.001		<0.001			<0.001		<0.001		<0.001	
***Cigarettes in past month*:**														
	n	% cigarettes (SE)	n	% cigarettes (SE)	n	% cigarettes (SE)		n	% cigarettes (SE)	n	% cigarettes (SE)	n	% cigarettes (SE)	
All years	50,034	7.3 (0.3)	2,094	41.2 (1.6)	47,940	5.7 (0.2)	*	50,977	9.5 (0.3)	3,183	43.7 (1.4)	47,794	7.1 (0.3)	*
2011	9,315	9.7 (0.8)	69	73.9 (8.1)	9,246	9.3 (0.7)	*	9,284	12.2 (0.8)	130	73.7 (5.2)	9,154	11.1 (0.7)	*
2012	12,275	8.1 (0.6)	180	78.9 (5.0)	12,095	7.0 (0.5)	*	12,369	11.1 (0.7)	342	74.2 (3.9)	12,027	9.2 (0.6)	*
2013	9,177	7.9 (0.6)	213	64.8 (5.6)	8,964	6.4 (0.5)	*	9,216	9.6 (0.7)	332	65.4 (4.5)	8,884	7.4 (0.6)	*
2014	10,645	5.4 (0.4)	849	37.1 (2.3)	9,796	2.7 (0.3)	*	11,150	7.5 (0.6)	1,183	38.3 (2.2)	9,967	4.0 (0.5)	*
2015	8,622	5.4 (0.5)	783	31.5 (2.4)	7,839	2.8 (0.4)	*	8,958	7.2 (0.7)	1,196	32.3 (2.5)	7,762	3.5 (0.6)	*
Trend^2^		<0.001		<0.001		<0.001			<0.001		<0.001		<0.001	
***Non-cigarette combustibles***^***3***^ ***in past month*:**														
	n	% combustible (SE)	n	% combustible (SE)	n	% combustible (SE)		n	% combustible (SE)	n	% combustible (SE)	n	% combustible (SE)	
All years	50,034	6.1 (0.2)	2,094	30.5 (1.5)	47,940	5.0 (0.2)	*	50,977	10.5 (0.3)	3,183	43.7 (1.3)	47,794	8.2 (0.3)	*
2011	9,315	6.9 (0.4)	69	46.3 (9.2)	9,246	6.6 (0.4)	*	9,284	12.4 (0.6)	130	66.8 (5.9)	9,154	11.4 (0.5)	*
2012	12,275	7.3 (0.5)	180	53.2 (4.9)	12,095	6.6 (0.4)	*	12,369	12.2 (0.6)	342	67.6 (4.8)	12,027	10.6 (0.5)	*
2013	9,177	7.5 (0.5)	213	47.6 (6.2)	8,964	6.5 (0.5)	*	9,216	11.6 (0.6)	332	62.3 (3.6)	8,884	9.6 (0.6)	*
2014	10,645	4.4 (0.4)	849	27.2 (2.3)	9,796	2.4 (0.3)	*	11,150	8.0 (0.6)	1,183	37.5 (2.4)	9,967	4.7 (0.5)	*
2015	8,622	4.7 (0.5)	783	24.5 (2.3)	7,839	2.7 (0.4)	*	8,958	8.3 (0.6)	1,196	35.7 (2.3)	7,762	4.3 (0.6)	*
Trend^2^		<0.001		<0.001		<0.001			<0.001		<0.001		<0.001	
***Smokeless tobacco***^***4***^ ***in past month*:**														
	n	% smokeless (SE)	n	% smokeless (SE)	n	% smokeless (SE)		n	% smokeless (SE)	n	% smokeless (SE)	n	% smokeless (SE)	
All years	50,034	1.8 (0.1)	2,094	12.9 (1.1)	47,940	1.3 (0.1)	*	50,977	7.6 (0.3)	3,183	31.8 (1.6)	47,794	5.9 (0.3)	*
2011	9,315	2.1 (0.2)	69	26.0 (8.3)	9,246	2.0 (0.2)		9,284	9.1 (0.9)	130	51.4 (6.7)	9,154	8.3 (0.8)	*
2012	12,275	2.2 (0.2)	180	28.7 (4.9)	12,095	1.8 (0.2)	*	12,369	8.2 (0.6)	342	45.4 (4.1)	12,027	7.1 (0.6)	*
2013	9,177	1.8 (0.2)	213	19.8 (4.9)	8,964	1.3 (0.2)	*	9,216	6.7 (0.8)	332	29.2 (3.6)	8,884	5.8 (0.7)	*
2014	10,645	1.7 (0.2)	849	11.1 (1.4)	9,796	0.9 (0.2)	*	11,150	7.2 (0.7)	1,183	31.7 (2.9)	9,967	4.5 (0.6)	*
2015	8,622	1.5 (0.3)	783	9.6 (1.4)	7,839	0.7 (0.2)	*	8,958	6.8 (0.9)	1,196	27.3 (2.5)	7,762	3.8 (0.7)	*
Trend^2^		0.030		<0.001		<0.001			0.035		<0.001		<0.001	
***Hookah in past month*:**														
	n	% hookah (SE)	n	% hookah (SE)	n	% hookah (SE)		n	% hookah (SE)	n	% hookah (SE)	n	% hookah (SE)	
All years	50,034	4.1 (0.2)	2,094	35.0 (2.2)	47,940	2.6 (0.1)	*	50,977	4.4 (0.2)	3,183	30.0 (1.6)	47,794	2.7 (0.1)	*
2011	9,315	2.5 (0.3)	69	21.1 (8.3)	9,246	2.3 (0.3)		9,284	3.2 (0.4)	130	32.2 (6.5)	9,154	2.7 (0.3)	*
2012	12,275	3.0 (0.3)	180	28.1 (4.6)	12,095	2.6 (0.3)	*	12,369	4.2 (0.4)	342	37.6 (4.2)	12,027	3.2 (0.3)	*
2013	9,177	3.4 (0.3)	213	28.8 (5.1)	8,964	2.7 (0.3)	*	9,216	3.6 (0.3)	332	29.7 (3.4)	8,884	2.6 (0.3)	*
2014	10,645	6.7 (0.5)	849	38.9 (3.5)	9,796	3.9 (0.4)	*	11,150	6.1 (0.5)	1,183	31.2 (2.8)	9,967	3.3 (0.3)	*
2015	8,622	4.8 (0.4)	783	34.9 (3.6)	7,839	1.7 (0.3)	*	8,958	5.0 (0.4)	1,196	27.5 (2.5)	7,762	1.7 (0.3)	*
Trend^2^		<0.001		0.108		0.913			<0.001		0.100		0.074	

As e-cigarette past month use increased, use became less exclusively associated with combustible tobacco use.

1. Chi-square test for difference in use of other tobacco products between past 30-day e-cigarette users and non-users (* = P < 0.001)

2. P-for-linear trend, 2011–2015

3. Non-cigarette combustibles include cigars, pipes, bidis, and kreteks (2011–2013)

4. Smokeless tobacco includes conventional smokeless tobacco, snus, and dissolvable tobacco

Marginal prevalences adjusted across years for grade in school and race/ethnicity

Subgroup sample sizes (n) are the mean sample size over 10 imputations for missing value assignment.

Abbreviations: n = sample size (denominator); SE = standard error

Among adolescents overall, past month use of other tobacco (≥1 product, excluding e-cigarettes) declined 2.8-percentage points (females) and 4.4-percentage points (males) during the 5-year period (Table 2). Over the same period, in the total adolescent population, past month use of e-cigarettes exclusively (no past month use of other tobacco products) increased 3.8-percentage points among females and 4.6-percentage points among males (not shown in tables).

### Trends in cigarette-related behaviors and intentions according to past month e-cigarette use

Over the study period and for both sexes, there was a tendency for past month cigarette users who also used e-cigarettes in the past month to be more likely to smoke cigarettes on all 30 days in the past month and to smoke >10 cigarettes per day, but only the association with daily smoking reached the P<0.001 threshold for statistical significance and only among males in 2011, 2012, and pooled over all five years (Table 3). As past month e-cigarette use became more prevalent from 2011–2015, there was a declining trend (statistically significant for males only) in daily smoking and smoking >10 cigarettes per day the percentage among past month e-cigarette users. Yet, among all past month cigarette users (i.e., not stratified by past month e-cigarette use), there was no statistically significant decline in cigarette smoking intensity during this period of rising e-cigarette past month use (Table 3).

**Table 3:** Cigarette use intensity and quit intentions according to past month use (yes or no) of electronic cigarettes, 2011–2015.

	Females, grades 6–12							Males, grades 6–12						
	All past 30-day cigarette users (n: sample size)		Concurrent past 30-day e-cigarette use				^1^	All past 30-day cigarette users (n: sample size)		Concurrent past 30-day e-cigarette use				^1^
			Yes (n: sample size)		No (n: sample size)					Yes (n: sample size)		No (n: sample size)		
***Cigarettes all 30 days in past month*:**														
	n	% all 30 (SE)	n	% all 30 (SE)	n	% all 30 (SE)		n	% all 30 (SE)	n	% all 30 (SE)	n	% all 30 (SE)	
All years	3,633	20.2 (0.9)	886	25.8 (2.2)	2,749	18.4 (1.2)		4,942	23.8 (0.9)	1,458	29.2 (1.7)	3,484	21.5 (1.1)	*
2011	893	21.2 (1.9)	-^2^	-	847	20.2 (2.0)		1,165	26.3 (1.8)	93	53.0 (7.2)	1,072	23.2 (1.9)	*
2012	970	20.4 (1.8)	145	32.5 (6.8)	825	18.3 (2.0)		1,349	23.7 (1.7)	260	38.8 (4.9)	1,089	20.2 (2.0)	*
2013	740	22.8 (2.3)	143	35.0 (5.5)	597	19.5 (2.9)		894	25.1 (2.1)	230	27.7 (4.6)	664	24.2 (2.9)	
2014	577	20.2 (2.2)	308	24.0 (3.3)	269	16.0 (2.9)		872	22.0 (2.2)	470	26.0 (3.6)	402	17.6 (3.2)	
2015	453	16.6 (2.2)	244	41.0 (3.2)	211	13.6 (2.9)		662	21.4 (2.2)	405	22.8 (2.8)	257	19.2 (4.3)	
Trend^3^		0.281		0.008		0.131			0.093		<0.001		0.322	
***Smoked >10 cigarettes/day*:**														
	n	% 10/day (SE)	n	% 10/day (SE)	n	% 10/day (SE)		n	% 10/day (SE)	n	% 10/day (SE)	n	% 10/day (SE)	
All years	3,633	6.4 (0.5)	886	9.9 (1.6)	2,749	5.2 (0.6)		4,942	12.4 (0.6)	1,458	16.3 (1.5)	3,484	10.8 (0.8)	
2011	893	6.1 (0.9)	-^2^	-	847	5.8 (0.9)		1,165	13.4 (1.3)	93	36.4 (7.7)	1,072	10.8 (1.2)	
2012	970	6.5 (1.1)	145	9.5 (4.0)	825	6.0 (1.2)		1,349	13.8 (1.3)	260	20.1 (3.7)	1,089	12.5 (1.5)	
2013	740	8.2 (1.3)	143	16.8 (5.8)	597	6.1 (1.9)		894	13.9 (1.4)	230	17.5 (4.0)	664	12.5 (1.9)	
2014	577	7.9 (1.3)	308	10.6 (2.1)	269	4.6 (1.6)		872	10.9 (2.0)	470	13.7 (3.4)	402	7.7 (2.9)	
2015	453	4.7 (1.1)	244	6.7 (2.1)	211	2.5 (1.3)		662	10.0 (1.0)	405	11.2 (2.0)	257	8.4 (2.2)	
Trend^3^		0.761		0.212		0.258			0.047		<0.001		0.274	
***Cigarette quit attempt in past year*:**														
	n	% quit attempt (SE)	n	% quit attempt (SE)	n	% quit attempt (SE)		n	% quit attempt (SE)	n	% quit attempt (SE)	n	% quit attempt (SE)	
All years	3,633	61.1 (1.2)	886	64.1 (2.9)	2,749	60.1 (1.5)		4,942	58.1 (1.0)	1,458	57.4 (2.0)	3,484	58.4 (1.3)	
2011	893	59.5 (2.4)	-^2^	-	847	60.0 (2.6)		1,165	57.7 (2.3)	93	57.4 (6.9)	1,072	57.7 (2.4)	
2012	970	61.7 (2.3)	145	60.6 (6.3)	825	61.9 (2.3)		1,349	55.7 (2.0)	260	51.5 (5.8)	1,089	56.7 (2.4)	
2013	740	57.8 (2.6)	143	56.4 (6.2)	597	58.1 (3.0)		894	58.8 (2.2)	230	56.0 (4.4)	664	60.3 (2.8)	
2014	577	61.6 (2.8)	308	62.1 (3.9)	269	60.4 (4.6)		872	61.4 (2.7)	470	58.2 (3.7)	402	64.4 (4.5)	
2015	453	66.6 (3.5)	244	73.1 (4.3)	211	57.7 (5.5)		662	58.0 (3.1)	405	58.8 (4.3)	257	55.2 (5.1)	
Trend^3^		0.194		0.023		0.618			0.370		0.509		0.500	
***Thinking about quitting cigarettes*:**														
	n	% think quit (SE)	n	% think quit (SE)	n	% think quit (SE)		n	% think quit (SE)	n	% think quit (SE)	n	% think quit (SE)	
All years	3,633	58.6 (1.4)	886	59.6 (2.5)	2,749	58.2 (1.7)		4,942	58.4 (1.1)	1,458	59.0 (2.0)	3,484	58.1 (1.4)	
2011	893	52.7 (3.2)	-^2^	-	847	52.9 (3.3)		1,165	47.8 (2.5)	93	48.7 (9.0)	1,072	47.8 (2.7)	
2012	970	64.6 (2.0)	145	62.6 (5.9)	825	65.1 (2.1)		1,349	64.7 (1.8)	260	55.4 (5.1)	1,089	67.6 (2.2)	
2013	740	55.0 (3.2)	143	49.4 (6.8)	597	56.4 (3.7)		894	57.8 (2.6)	230	56.6 (5.3)	664	58.4 (3.1)	
2014	577	61.7 (2.6)	308	61.1 (3.7)	269	61.9 (3.8)		872	63.3 (2.6)	470	60.6 (3.5)	402	65.6 (4.8)	
2015	453	61.8 (3.4)	244	63.0 (4.6)	211	60.3 (4.4)		662	61.5 (3.3)	405	62.4 (3.6)	257	59.7 (5.4)	
Trend^3^		0.129		0.372		0.233			0.003		0.111		0.009	
***Thinking about quitting all tobacco*:**														
	n	% think quit (SE)	n	% think quit (SE)	n	% think quit (SE)		n	% think quit (SE)	n	% think quit (SE)	n	% think quit (SE)	
All years	3,633	53.9 (1.3)	886	48.3 (2.6)	2,749	56.0 (1.6)		4,942	48.2 (1.1)	1,458	45.4 (2.1)	3,484	49.5 (1.3)	
2011	893	54.7 (2.9)	-^2^	-	847	54.7 (2.9)		1,165	46.3 (1.9)	93	49.5 (7.2)	1,072	46.1 (2.1)	
2012	970	60.2 (2.5)	145	54.5 (6.6)	825	61.1 (2.6)		1,349	54.3 (2.0)	260	50.5 (5.3)	1,089	55.5 (2.4)	
2013	740	52.1 (3.0)	143	47.9 (6.3)	597	53.5 (3.7)		894	50.6 (2.9)	230	49.0 (5.3)	664	51.5 (3.2)	
2014	577	44.4 (2.8)	308	40.7 (4.3)	269	49.7 (4.7)		872	43.9 (3.0)	470	41.9 (3.5)	402	46.1 (4.9)	
2015	453	55.0 (3.9)	244	52.3 (4.1)	211	57.8 (5.4)		662	43.3 (3.1)	405	42.3 (4.1)	257	44.6 (4.5)	
Trend^3^		0.155		0.476		0.766			0.135		0.101		0.943	

Among past 30-day cigarette users, past 30-day e-cigarette use was not associated with cigarette quit attempts or quit intentions.

1. Chi-square test for difference in cigarette smoking intensity or quit behavior/intentions between past 30-day cigarette users with or without past 30-day e-cigarette use (* P < 0.001)

2. Estimates suppressed due to sample size (n < 50)

3. P-for-linear trend, 2011–2015

Marginal percentages adjusted across years for grade in school and race/ethnicity

Subgroup sample sizes (n) are the mean sample size over 10 imputations for missing value assignment.

Abbreviations: n = sample size (denominator); SE = standard error

As shown in Table 3, among all past month cigarette users, there was no statistically significant linear trend in the percentage that made a cigarette quit attempt in the past year, reported thinking about quitting cigarettes, or reported thinking about quitting all tobacco In all years and both sexes, among past 30-day cigarette users, there was no association between past month e-cigarette use and past-year cigarette quit attempts, thinking about quitting cigarettes, or thinking about quitting all tobacco (Table 3).

### Sensitivity checks

Results were largely unchanged under sensitivity analyses based on complete cases (Tables 1–3 in S2 File). Restricted to 2011–2013 data, statistically significant trends in increasing e-cigarette ever use and past month use were maintained (Table 4 in S2 File). The trends showing lower prevalence of past month use of other tobacco products among past month e-cigarette users remained in the same direction but were less pronounced and not consistently statistically significant when 2014 and 2015 data were excluded (Table 5 in S2 File). There remained no association between past month e-cigarette use and past year cigarette quit attempts or cigarette and all tobacco quit contemplation in the 2011–2013 data (Table 6 in S2 File).

## Discussion

During a period of rapidly increasing e-cigarette use (past month and ever), the average profile of the adolescent e-cigarette using population (past month and ever) became less predominantly male, less singularly associated with use of other tobacco products, and became increasingly likely to include individuals who had never tried or did use other tobacco products in the past month. Expanding e-cigarette ever and past month use occurred simultaneously with a demonstrable shift in youth consumption of other tobacco products. Although past month combustible tobacco use declined, more U.S. middle school and high school students reported past month use of some form of tobacco and/or e-cigarettes in 2014–2015 than in 2011–2013, mostly driven by increasing past month use of e-cigarettes and hookah, as reported elsewhere [6]. Although e-cigarette past month users were less likely to be past month multi-product users in 2014–2015 than in previous years (2011–2013), in all years and both sexes, past month e-cigarette users were several times more likely than past month e-cigarette non-users also to use one or more other tobacco product in the past month, and those who also smoked cigarettes in the past month were not more likely to have attempted or contemplated quitting.

Meaningful decreases in adolescent past month cigarette use from 2011–2015 overlapped with large increases in past month e-cigarette use, raising the possibility of product substitution. It is plausible that both replacement (i.e., adolescents' past month use of e-cigarettes instead of cigarettes and other tobacco products) and expansion (i.e., ever or past month use of e-cigarettes by adolescents who would not otherwise use tobacco) took place from 2011–2015. Over the five-year period, the absolute overall decline in past month use of all non e-cigarette tobacco products was nearly as large as the absolute overall increase in past month e-cigarette use.

The extent to which each of these two possibilities (substitution or expansion) accounts for the observed shifts in behavior cannot be estimated from these data. The observed declines in cigarette smoking were not unprecedented in the history of the NYTS. From 2000–2006, past month cigarette smoking by U.S. high school students declined from 28.0% to 19.8% [26]. Therefore, the recent availability e-cigarettes occurred during an existing trend of declining youth cigarette use. It is not knowable to what extent the decline in adolescent smoking from 2011–2015 represents a shift from what would have occurred had e-cigarettes not been available. Further research into adolescents' motivations for using e-cigarettes, potentially in lieu of other tobacco products, is recommended.

The long-term public health effect of excess youth e-cigarette past month use cannot be quantified presently, but would be particularly destructive if e-cigarette past month use encourages, rather than replaces, other tobacco use. E-cigarette use is positively associated with cigarette smoking intention among adolescent non-smokers [27,28], and recent prospective studies have reported that non-smoking adolescents [29–31] and young adults [32] who use e-cigarettes are more likely to initiate future cigarette use.

### Multi-product use

Multi-product tobacco use in adolescence carries over into adulthood. Over 80% of dual- users of smokeless tobacco and cigarettes in adolescence continued dual-use as young adults [33]. In the present study, over 50% of adolescents who reported past month use of at least one other tobacco product in 2015 were also past month users of e-cigarettes. Long-term data do not yet exist to track past month use of both e-cigarettes and other tobacco from adolescence into adulthood. However, whether e-cigarette ever and past month use has lasting implications for use of other tobacco products bears critical implications for the net population health impact of rising e-cigarette use in the overall population presently and in future years [1,4].

In the present study, the percentage of past month e-cigarette users who also used other tobacco products in the past month was lower in 2015 than in 2011. This finding must be interpreted cautiously. This result does not imply a static population of past month e-cigarette users. Rather, the proportion with past month use of other products was calculated amid a growing denominator of e-cigarette past month users each consecutive year. In other words, past month e-cigarette use increasingly encompassed a broader population segment, capturing individuals who in previous years would have been past month e-cigarette non-users. As past month e-cigarette use became more common, the population who used e-cigarettes comprised more past month non-users of other tobacco products.

It is plausible that at least some individuals used e-cigarettes in the past month in place of or after switching from other tobacco products. As discussed above, the present analysis cannot determine directly whether the decrease in past month use of other tobacco products is a continuation of an existing trend that would have occurred without the emergence of e-cigarettes. It is also unknown to what extent the growing population of youth who had only used e-cigarettes in the past month now includes adolescents who otherwise would have been other tobacco product users or non-users. Further research is warranted to assess the extent to which e-cigarettes might attract new populations of individuals to nicotine-containing products versus acting as an alternative to cigarettes and other tobacco products.

### Cigarette use intensity

Among cigarette smokers, past 30-day e-cigarette use was not statistically significantly associated with daily smoking or cigarettes smoked per day, with the exception of a significantly higher percentage of daily smoking among male past month e-cigarette users in 2011 and 2012. In Wales, adolescent heavy smokers were more likely to have bought or tried e-cigarettes than light smokers [34], as was the case in Poland, where dual-use of cigarettes and e-cigarettes was associated with more frequent smoking and greater cigarette consumption [19]. It is possible that cigarette users in these studies turned to e-cigarettes as a means to reduce their smoking, but the temporal ordering between e-cigarette initiation and smoking intentions in those studies or the present analysis is not clear. In the present study, the overall prevalence of intense cigarette use did not decline to a statistically significant extent during this period of declining smoking prevalence and rising e-cigarette use. We speculate that the observed decline in intensity of cigarette use among male past 30-day cigarette users who also used e-cigarettes in the past 30-days could reflect expanding past month e-cigarette use among less intense cigarette users after a period of early adoption primarily among heavier smokers.

### E-cigarette use and smoking quit attempts or contemplation

Over the study period, there was no consistently statistically significant trend in past month cigarette smokers reporting quit attempts or thinking about quitting cigarette use. Quitting contemplation was not associated with past month e-cigarette use in any survey year, consistent with results from previous cross-sectional studies of U.S. high school or college students [20,35,36]. Likewise, in a sample of high school students in Canada, current cigarette smokers who had ever tried to quit smoking were not more likely to have tried e-cigarettes than never-quitters [15]. In the present study, whether adolescents purposefully turned to e-cigarettes for smoking cessation versus other reasons was not specifically measured, but the lack of positive associations between e-cigarette past month use and quitting contemplation or quit attempts suggests that e-cigarette initiation is not taking place in larger numbers among adolescents looking to quit cigarettes than among those without an intention to quit. In surveys completed in Switzerland and the U.S., adolescents cited curiosity far more frequently than smoking reduction as a reason for e-cigarette use [37,38]. This differs from findings for adult e-cigarette initiators, who cite smoking cessation as a motivation more commonly, especially among current smokers [39]. A systematic review of two randomized controlled trials in adults concluded that e-cigarettes are superior to placebo e-cigarettes and not significantly different from the nicotine patch in helping smokers to quit but graded the evidence as "low" or "very low" overall quality due to the small number of trials [40]. In population-based observational studies, e-cigarette use has been associated with less successful cigarette quitting among adults [41] and adolescents [20,21].

The present study did not include a measure of the type or generation of e-cigarette devices being used. In the 2015 NYTS, more than half (53.4%) of e-cigarette ever users reported using only rechargeable or refillable devices [42], but device type was not measured in earlier waves. It is possible that earlier and later generation devices may differ in their associations with ever and past month use of other tobacco products and with cigarette quit attempts or quit contemplation among youth. Whether and how trends in these associations might differ by e-cigarette type are questions worthy of tracking in future NYTS waves.

There was also no statistically significant trend in reporting thinking about quitting all tobacco. The survey did not assess which non-cigarette tobacco products adolescents were considering quitting or continuing, or their specific motivations. Other studies suggest that adolescents or young adults view e-cigarettes, hookah, and other alternative tobacco products as less harmful than cigarettes [43–45], particularly dual-users with cigarettes [43,46], which might contribute to their motivation to quit all tobacco completely.

### Limitations and alternative interpretations

Although NYTS data were collected over five consecutive years, each survey wave was administered to a separate cross-sectional population; therefore, the ordering between e-cigarette initiation and other tobacco behaviors could not be observed for individuals. It is possible that tobacco susceptible adolescents were independently predisposed to use both e-cigarettes and other tobacco products. Confounding variables were not taken into account in the analysis. Therefore, without the ability to rule out confounding or to establish the temporal relationship between quit attempts, past month e-cigarette use, and past month cigarette use, causal conclusions cannot be drawn from these associations alone.

In addition to possible confounding and the repeated cross sectional design, other study limitations warrant consideration. National data may obscure potentially important differences in e-cigarette use by region or rural/urban subpopulations, and individuals not enrolled in schools (e.g. home schooled or institutionalized adolescents) were excluded from the sample.

In terms of measurement, use of multiple products within the previous month does not necessarily indicate routine, regular, or heavy use of multiple products, and could indicate concurrent recent experimentation [18]. Use of ≥2 tobacco/nicotine products in the prior 30 days is common among adolescents who use tobacco products only 1 to 5 days per month, including among the majority of infrequent users of e- cigarettes [47]. However, while past month use may not indicate frequent use, past month use of multiple products among adolescents and young adults has been associated with psychosocial characteristics and behaviors, including use of marijuana, alcohol, and other drugs [48], sensation seeking, earlier age of tobacco initiation [49], and positive attitudes about tobacco companies and tobacco products [50]. Future research into the use of more than one tobacco product within a given time period is needed to improve our understanding of these behaviors and their implications for tobacco use trajectories among youth.

Categorizing tobacco behavior according to past month use will include both frequent and infrequent tobacco users in the same category. Decades of research on cigarette smoking support past month behavior as the standard measure for adolescent tobacco surveillance. Adolescents are much less likely to smoke daily than adults [51], but young non-daily smokers are at elevated risk of progression to daily smoking in adulthood [52]. Symptoms of tobacco dependence have been reported among adolescents who use tobacco products only 1 to 2 days in the prior month [53]. Past month smoking, even infrequently, is predictive of future smoking. Recent analysis of the US National Longitudinal Study of Adolescent to Adult Health showed that low intensity non-daily smoking in adolescence (smoked 1–5 cigarettes in the past month) was strongly associated with smoking in adulthood 14 years later [54].

As with cigarettes, most adolescent past month e-cigarette users do not use e-cigarettes daily, and many use infrequently [47]. Long-term, longitudinal evidence is not yet available regarding the outcomes of infrequent e-cigarette use by youth. Infrequent, recent use of other non-cigarette tobacco products is associated with future outcomes. For example, ever use of smokeless tobacco by self-identified non-regular users was strongly associated with progression to regular use over 4 years [55]. Among high school cigarette never smokers, past month e-cigarette use has been shown to be associated with cigarette smoking susceptibility [56] and future cigarette initiation [57], including in meta-analysis [58]. More research is essential for understanding the long-term implication of frequent and infrequent e-cigarette use by youth.

Phrasing of the e-cigarette use items changed during the study time period, potentially affecting comparability across survey waves. The format of the item recording past month e-cigarette use changed in 2014, with the e-cigarette item made more consistent with items for cigarettes and smokeless tobacco. This change may have contributed to the rise in reported e-cigarette prevalence between 2013 and 2014. E-cigarette past month use continued to increase from 2014 to 2015 under the new questionnaire format, as reported elsewhere [42]. However, the 2014 e-cigarette items did not include alternative e-cigarette terminology, such as “vapor pens” or “e-hookah,” which could have underestimated e-cigarette use [59] relative to 2015, when these terms were added. Despite questionnaire changes, large increases in youth e-cigarette ever and past month use over this time period were mirrored in other national surveillance [7]. In a sensitivity analyses that excluded the 2014 and 2015 NYTS dataset, virtually all trends maintained the same direction as in the full dataset, although not all trends remained statistically significant, potentially due to lost statistical power under the lower prevalence of past month e-cigarette use prior to 2014. Additionally, the response choice order for several other tobacco products, including hookah, pipes, and bidis, differed from year-to-year, which may have contributed to variation in the estimated prevalence of past month use of other tobacco products. For example, prevalence of past month hookah use was highest in 2014, when hookah was listed first among response options. As a final consideration on variable definitions, the binary quit contemplation variables used in this analysis combined both individuals planning to quit in the near future and those planning to quit but not within the next 12 months. While most respondents did plan to quit within 12 months, it is conceivable that associations with e-cigarettes could differ depending on the planned timeframe for quitting.

Advantageously, this study included a large sample collected under a rigorous protocol and evaluated using a tested survey instrument. Of note, prevalence values were estimated using multiple imputation to address plausible non-response bias and may differ from other reports based on complete-case NYTS data.

### Regulatory implications

Any proposed e-cigarette regulatory action must consider implications for adolescents, which may differ importantly from those for adults. New regulatory measures should be considered within the context of temporal changes in the characteristics of the e-cigarette using population (including ever and month past users).

## Conclusions

Over the five-year period from 2011–2015, adolescent e-cigarette ever and past month use transitioned from an activity predominated by males and past month users of multiple tobacco products to more mainstream behavior that increasingly included never-users of cigarettes and other tobacco products. The rise in e-cigarette popularity correlated with a sharp, albeit not unprecedented, decline in past month youth cigarette smoking, which could coincide with e-cigarette substitution for cigarettes among some youth. However, e-cigarette past month use, as measured in this analysis, was not associated with increased smoking quit attempts or quit contemplation, and the percentage of adolescents using any tobacco product and/or e-cigarettes remained high through 2015, suggesting there is also the potential for past month e-cigarette use among youth who would not otherwise have used cigarettes or other tobacco.

## Supporting information

S1 File Past 30-day electronic cigarette use by race/ethnicity, 2011–2015. (PDF)

S2 File Sensitivity analyses: Complete case approach and results restricted to 2011–2013. (PDF)

## References

[1] Schneider S, Diehl K. Vaping as a catalyst for smoking? An initial model on the initiation of electronic cigarette use and the transition to tobacco smoking among adolescents. Nicotine Tob Res. 2016;18: 647–653. doi: 10.1093/ntr/ntv193 26386472 10.1093/ntr/ntv193

[2] Cobb CO, Villani AC, Graham AL, Pearson JL, Glasser AM, Rath JM, et al Markov modeling to estimate the population impact of emerging tobacco products: A proof-of-concept study. Tob Regul Sci. 2015;1: 129–141.

[3] Benowitz NL. Emerging nicotine delivery products. Implications for public health. Ann Am Thorac Soc. 2014;11: 231–235. doi: 10.1513/AnnalsATS.201312-433PS 24575992 10.1513/AnnalsATS.201312-433PS

[4] Kalkhoran S, Glantz SA. Modeling the Health Effects of Expanding e-Cigarette Sales in the United States and United Kingdom: A Monte Carlo Analysis. JAMA Intern Med. 2015;175: 1671–1680. doi: 10.1001/jamainternmed.2015.4209 26322924 10.1001/jamainternmed.2015.4209 PMC4594196

[5] Lauterstein D, Hoshino R, Gordon T, Watkins BX, Weitzman M, Zelikoff J. The changing face of tobacco use among United States youth. Curr Drug Abuse Rev. 2014;7: 29–43. 25323124 10.2174/1874473707666141015220110 PMC4469045

[6] Arrazola RA, Singh T, Corey CG, Husten CG, Neff LJ, Apelberg BJ, et al Tobacco use among middle and high school students—United States, 2011–2014. MMWR Morb Mortal Wkly Rep. 2015;64: 381–385. 25879896 PMC5779546

[7] Johnston LD, O'Malley PM, Miech RA, Bachman JG, Schulenberg JE. Monitoring the future, national survey results on drug use: 1975–2014: Overview, key findings on adolescent drug use. Ann Arbor, MI: Institute for Social Research, University of Michigan; 2015. Available: http://www.monitoringthefuture.org/pubs/monographs/mtf-overview2014.pdf

[8] de Andrade M, Hastings G, Angus K. Promotion of electronic cigarettes: Tobacco marketing reinvented? BMJ. 2013;347: f7473 doi: 10.1136/bmj.f7473 24361526 10.1136/bmj.f7473

[9] Corey CG, Ambrose BK, Apelberg BJ, King BA. Flavored tobacco product use among middle and high school students—United States, 2014. MMWR Morb Mortal Wkly Rep. 2015;64: 1066–1070. doi: 10.15585/mmwr.mm6438a2 26421418 10.15585/mmwr.mm6438a2

[10] Hildick-Smith GJ, Pesko MF, Shearer L, Hughes JM, Chang J, Loughlin GM, et al A practitioner's guide to electronic cigarettes in the adolescent population. J Adolesc Health. 2015;57: 574–579. doi: 10.1016/j.jadohealth.2015.07.020 26422289 10.1016/j.jadohealth.2015.07.020

[11] Walley SC, Jenssen BP, American Academy of Pediatrics Section on Tobacco Control. Electronic nicotine delivery systems. Pediatrics. 2015;136: 1018–1026. doi: 10.1542/peds.2015-3222 26504128 10.1542/peds.2015-3222

[12] Stanwick R. E-cigarettes: Are we renormalizing public smoking? Reversing five decades of tobacco control and revitalizing nicotine dependency in children and youth in Canada. Paediatr Child Health. 2015;20: 101–105. 25838785 10.1093/pch/20.2.101 PMC4373571

[13] U.S. Food and Drug Administration. Deeming Tobacco Products To Be Subject to the Federal Food, Drug, and Cosmetic Act, as Amended by the Family Smoking Prevention and Tobacco Control Act; Restrictions on the Sale and Distribution of Tobacco Products and Required Warning Statements for Tobacco Products. 10 May 2016. Available from: https://www.federalregister.gov/documents/2016/05/10/2016-10685/deeming-tobacco-products-to-be-subject-to-the-federal-food-drug-and-cosmetic-act-as-amended-by-the. Cited 3 February 2017. 27192730

[14] Babineau K, Taylor K, Clancy L. Electronic cigarette use among Irish youth: A cross sectional study of prevalence and associated factors. PLoS One. 2015;10: e0126419 doi: 10.1371/journal.pone.0126419 26018542 10.1371/journal.pone.0126419 PMC4446031

[15] Czoli CD, Hammond D, Reid JL, Cole AG, Leatherdale ST. Use of conventional and alternative tobacco and nicotine products among a sample of Canadian youth. J Adolesc Health. 2015;57: 123–125. doi: 10.1016/j.jadohealth.2015.03.006 25937469 10.1016/j.jadohealth.2015.03.006

[16] Moore G, Hewitt G, Evans J, Littlecott HJ, Holliday J, Ahmed N, et al Electronic-cigarette use among young people in Wales: Evidence from two cross-sectional surveys. BMJ Open. 2015;5: e007072-2014-007072. 10.1136/bmjopen-2014-007072 PMC4420942 25877272

[17] Wills TA, Knight R, Williams RJ, Pagano I, Sargent JD. Risk factors for exclusive e-cigarette use and dual e-cigarette use and tobacco use in adolescents. Pediatrics. 2015;135: e43–51. doi: 10.1542/peds.2014-0760 25511118 10.1542/peds.2014-0760 PMC4279062

[18] Villanti AC, Pearson JL, Glasser AM, Johnson AL, Collins LK, Niaura RS, Abrams DB3. Frequency of youth e-cigarette and tobacco use patterns in the U.S.: Measurement precision is critical to inform public health. Nicotine Tob Res. 2016 12 24. pii: ntw388. [Epub ahead of print] 10.1093/ntr/ntw388 PMC5896511 28013271

[19] Goniewicz ML, Leigh NJ, Gawron M, Nadolska J, Balwicki L, McGuire C, et al Dual use of electronic and tobacco cigarettes among adolescents: A cross-sectional study in Poland. Int J Public Health. 2016;61: 189–197. doi: 10.1007/s00038-015-0756-x 26521213 10.1007/s00038-015-0756-x

[20] Dutra LM, Glantz SA. Electronic cigarettes and conventional cigarette use among U.S. adolescents: A cross-sectional study. JAMA Pediatr. 2014;168: 610–617. doi: 10.1001/jamapediatrics.2013.5488 24604023 10.1001/jamapediatrics.2013.5488 PMC4142115

[21] Lee S, Grana RA, Glantz SA. Electronic cigarette use among korean adolescents: A cross-sectional study of market penetration, dual use, and relationship to quit attempts and former smoking. J Adolesc Health. 2014;54: 684–690. doi: 10.1016/j.jadohealth.2013.11.003 24274973 10.1016/j.jadohealth.2013.11.003 PMC4031306

[22] Office on Smoking and Health. 2014 National Youth Tobacco Survey: Methodology Report. Atlanta, GA: U.S. Department of Health and Human Services, Centers for Disease Control and Prevention, National Center for Chronic Disease Prevention and Health Promotion, Office on Smoking and Health, 2015. Available: http://www.cdc.gov/tobacco/data_statistics/surveys/nyts/

[23] von Elm E, Altman DG, Egger M, Pocock SJ, Gøtzsche PC, Vandenbroucke JP, et al The Strengthening the Reporting of Observational Studies in Epidemiology (STROBE) statement: guidelines for reporting observational studies. Lancet. 2007;370: 1453–1457. doi: 10.1016/S0140-6736(07)61602-X 18064739 10.1016/S0140-6736(07)61602-X

[24] Rubin DB. The calculation of posterior distributions by data augmentation: Comment: A noniterative sampling/importance resampling alternative to the data augmentation algorithm for creating a few imputations when fractions of missing information are modest: The SIR algorithm. J Am Stat Assoc. 1987;82: 543–546.

[25] Witt MB, Spagnola KE. Using predictive marginals to produce standardized estimates. In: Proceedings of the Survey Research Methods Section, American Statistical Association. 2009. Available: https://www.amstat.org/sections/srms/proceedings/y2009/Files/305262.pdf

[26] Centers for Disease Control and Prevention (CDC). Tobacco use among middle and high school students—United States, 2000–2009. MMWR Morb Mortal Wkly Rep. 2010;59: 1063–1068. 20798668

[27] Bunnell RE, Agaku IT, Arrazola RA, Apelberg BJ, Caraballo RS, Corey CG, et al Intentions to smoke cigarettes among never-smoking US middle and high school electronic cigarette users: National Youth Tobacco Survey, 2011–2013. Nicotine Tob Res. 2015;17: 228–235. doi: 10.1093/ntr/ntu166 25143298 10.1093/ntr/ntu166 PMC4515756

[28] Wills TA, Sargent JD, Knight R, Pagano I, Gibbons FX. E-cigarette use and willingness to smoke: A sample of adolescent non-smokers. Tob Control. 2016;25: e52–e59. doi: 10.1136/tobaccocontrol-2015-052349 26261237 10.1136/tobaccocontrol-2015-052349 PMC4840020

[29] Leventhal AM, Strong DR, Kirkpatrick MG, Unger JB, Sussman S, Riggs NR, et al Association of electronic cigarette use with initiation of combustible tobacco product smoking in early adolescence. JAMA. 2015;314: 700–707. doi: 10.1001/jama.2015.8950 26284721 10.1001/jama.2015.8950 PMC4771179

[30] Primack BA, Soneji S, Stoolmiller M, Fine MJ, Sargent JD. Progression to traditional cigarette smoking after electronic cigarette use among US adolescents and young adults. JAMA Pediatr. 2015;169: 1018–1023. doi: 10.1001/jamapediatrics.2015.1742 26348249 10.1001/jamapediatrics.2015.1742 PMC4800740

[31] Wills TA, Knight R, Sargent JD, Gibbons FX, Pagano I, Williams RJ. Longitudinal study of e-cigarette use and onset of cigarette smoking among high school students in Hawaii. Tob Control. 2016. [Epub ahead of print] 10.1136/tobaccocontrol-2015-052705 PMC4959970 26811353

[32] Gmel G, Baggio S, Mohler-Kuo M, Daeppen JB, Studer J. E-cigarette use in young Swiss men: Is vaping an effective way of reducing or quitting smoking? Swiss Med Wkly. 2016;146: w14271 doi: 10.4414/smw.2016.14271 26752454 10.4414/smw.2016.14271

[33] Kaufman AR, Land S, Parascandola M, Augustson E, Backinger CL. Tobacco use transitions in the United States: The national longitudinal study of adolescent health. Prev Med. 2015;81: 251–257. doi: 10.1016/j.ypmed.2015.08.026 26361752 10.1016/j.ypmed.2015.08.026 PMC4679648

[34] Hughes K, Bellis MA, Hardcastle KA, McHale P, Bennett A, Ireland R, et al Associations between e-cigarette access and smoking and drinking behaviours in teenagers. BMC Public Health. 2015;15: 244-015-1618-4. 10.1186/s12889-015-1618-4 PMC4379746 25886064

[35] Sutfin EL, McCoy TP, Morrell HE, Hoeppner BB, Wolfson M. Electronic cigarette use by college students. Drug Alcohol Depend. 2013;131: 214–221. doi: 10.1016/j.drugalcdep.2013.05.001 23746429 10.1016/j.drugalcdep.2013.05.001 PMC3760168

[36] Lippert AM. Do adolescent smokers use E-cigarettes to help them quit? The sociodemographic correlates and cessation motivations of U.S. adolescent E-cigarette use. Am J Health Promot. 2015;29: 374–379. doi: 10.4278/ajhp.131120-QUAN-595 24968185 10.4278/ajhp.131120-QUAN-595

[37] Kong G, Morean ME, Cavallo DA, Camenga DR, Krishnan-Sarin S. Reasons for electronic cigarette experimentation and discontinuation among adolescents and young adults. Nicotine Tob Res. 2015;17: 847–854. doi: 10.1093/ntr/ntu257 25481917 10.1093/ntr/ntu257 PMC4674436

[38] Surís JC, Berchtold A, Akre C. Reasons to use e-cigarettes and associations with other substances among adolescents in Switzerland. Drug Alcohol Depend. 2015;153: 140–144. doi: 10.1016/j.drugalcdep.2015.05.034 26077606 10.1016/j.drugalcdep.2015.05.034

[39] Li J, Newcombe R, Walton D. The prevalence, correlates and reasons for using electronic cigarettes among New Zealand adults. Addict Behav. 2015;45: 245–251. doi: 10.1016/j.addbeh.2015.02.006 25744712 10.1016/j.addbeh.2015.02.006

[40] Hartmann-Boyce J, McRobbie H, Bullen C, Begh R, Stead LF, Hajek P. Electronic cigarettes for smoking cessation. Cochrane Database Syst Rev. 2016 9 14;9:CD010216 (Epub ahead of print). doi: 10.1002/14651858.CD010216.pub3 27622384 10.1002/14651858.CD010216.pub3 PMC6457845

[41] Kalkhoran S, Glantz SA. E-cigarettes and smoking cessation in real-world and clinical settings: A systematic review and meta-analysis. Lancet Respir Med. 2016;4: 116–128. doi: 10.1016/S2213-2600(15)00521-4 26776875 10.1016/S2213-2600(15)00521-4 PMC4752870

[42] Singh T, Arrazola RA, Corey CG, Husten CG, Neff LJ, Homa DM, et al Tobacco Use Among Middle and High School Students—United States, 2011–2015. MMWR Morb Mortal Wkly Rep. 2016;65: 361–367. doi: 10.15585/mmwr.mm6514a1 27077789 10.15585/mmwr.mm6514a1

[43] Latimer LA, Batanova M, Loukas A. Prevalence and harm perceptions of various tobacco products among college students. Nicotine Tob Res. 2014;16: 519–526. doi: 10.1093/ntr/ntt174 24212764 10.1093/ntr/ntt174

[44] Amrock SM, Zakhar J, Zhou S, Weitzman M. Perception of e-cigarette harm and its correlation with use among U.S. adolescents. Nicotine Tob Res. 2015;17: 330–336. doi: 10.1093/ntr/ntu156 25125321 10.1093/ntr/ntu156 PMC5479512

[45] Berg CJ, Stratton E, Schauer GL, Lewis M, Wang Y, Windle M, et al Perceived harm, addictiveness, and social acceptability of tobacco products and marijuana among young adults: Marijuana, hookah, and electronic cigarettes win. Subst Use Misuse. 2015;50: 79–89. doi: 10.3109/10826084.2014.958857 25268294 10.3109/10826084.2014.958857 PMC4302728

[46] Cooper M, Case KR, Loukas A, Creamer MR, Perry CL. E-cigarette Dual Users, Exclusive Users and Perceptions of Tobacco Products. Am J Health Behav. 2016;40: 108–116. doi: 10.5993/AJHB.40.1.12 26685819 10.5993/AJHB.40.1.12 PMC4869518

[47] Neff LJ, Arrazola RA, Caraballo RS, Corey CG, Cox S, King BA, et al Frequency of Tobacco Use Among Middle and High School Students—United States, 2014. MMWR Morb Mortal Wkly Rep. 2015;64: 1061–1065. doi: 10.15585/mmwr.mm6438a1 26422781 10.15585/mmwr.mm6438a1

[48] Creamer MR, Portillo GV, Clendennen SL, Perry CL. Is Adolescent Poly-tobacco Use Associated with Alcohol and Other Drug Use? Am J Health Behav. 2016;40: 117–1122. doi: 10.5993/AJHB.40.1.13 26685820 10.5993/AJHB.40.1.13 PMC4869866

[49] Soneji S, Sargent J, Tanski S. Multiple tobacco product use among US adolescents and young adults. Tob Control. 2016;25: 174–180. doi: 10.1136/tobaccocontrol-2014-051638 25361744 10.1136/tobaccocontrol-2014-051638 PMC4547881

[50] Kowitt SD, Patel T, Ranney LM, Huang LL, Sutfin EL, Goldstein AO. Poly-Tobacco Use among High School Students. Int J Environ Res Public Health. 2015;12: 14477–14489. doi: 10.3390/ijerph121114477 26580636 10.3390/ijerph121114477 PMC4661661

[51] Okuyemi KS, Harris KJ, Scheibmeir M, Choi WS, Powell J, Ahluwalia JS. Light smokers: issues and recommendations. Nicotine Tob Res. 2002;4 Suppl 2: S103–S112. 12573172 10.1080/1462220021000032726

[52] Robertson L, Iosua E, McGee R, Hancox RJ. Nondaily, Low-Rate Daily, and High-Rate Daily Smoking in Young Adults: A 17-Year Follow-Up. Nicotine Tob Res. 2016;18: 943–949. doi: 10.1093/ntr/ntv167 26246050 10.1093/ntr/ntv167 PMC5896816

[53] Apelberg BJ, Corey CG, Hoffman AC, Schroeder MJ, Husten CG, Caraballo RS, et al Symptoms of tobacco dependence among middle and high school tobacco users: results from the 2012 National Youth Tobacco Survey. Am J Prev Med. 2014;47(2 Suppl 1): S4–S14. 25044195 10.1016/j.amepre.2014.04.013 PMC4624110

[54] Saddleson ML, Kozlowski LT, Giovino GA, Homish GG, Mahoney MC, Goniewicz ML. Assessing 30-day quantity-frequency of U.S. adolescent cigarette smoking as a predictor of adult smoking 14 years later. Drug Alcohol Depend. 2016;162: 92–98. doi: 10.1016/j.drugalcdep.2016.02.043 26987520 10.1016/j.drugalcdep.2016.02.043 PMC6119624

[55] Tomar SL, Giovino GA. Incidence and predictors of smokeless tobacco use among US youth. Am J Public Health. 1998;88: 20–26. 9584028 10.2105/ajph.88.1.20 PMC1508391

[56] Barrington-Trimis JL, Berhane K, Unger JB, Cruz TB, Urman R, Chou CP, et al The E-cigarette Social Environment, E-cigarette Use, and Susceptibility to Cigarette Smoking. J Adolesc Health. 2016;59: 75–80. doi: 10.1016/j.jadohealth.2016.03.019 27161417 10.1016/j.jadohealth.2016.03.019 PMC4920702

[57] Barrington-Trimis JL, Urman R, Berhane K, Unger JB, Cruz TB, Pentz MA, et al E-Cigarettes and Future Cigarette Use. Pediatrics. 2016;138. pii: e20160379. 10.1542/peds.2016-0379 PMC4925085 27296866

[58] Zhong J, Cao S, Gong W, Fei F, Wang M. Electronic Cigarettes Use and Intention to Cigarette Smoking among Never-Smoking Adolescents and Young Adults: A Meta-Analysis. Int *J Environ Res Public Health*. 2016;13. pii: E465. 10.3390/ijerph13050465 PMC4881090 27153077

[59] Richtel M. E-Cigarettes, by Other Names, Lure Young and Worry Experts. New York Times. 4 March 2014. Available: http://www.nytimes.com/2014/03/05/business/e-cigarettes-under-aliases-elude-the-authorities.html?_r=0. Accessed April 22, 2016.

