# Supplementary material for: Trends in characteristics and multi-product use among adolescents who use electronic cigarettes, United States 2011-2015
Source: PLoS One. 2017 May 5;12(5):e0177073. doi: 10.1371/journal.pone.0177073 (PMC5419603; doi:10.1371/journal.pone.0177073)
Supplement: S1 File — (PDF) [file pone.0177073.s001.pdf]

S1 Table. Past 30-day electronic cigarette use according to race/ethnicity, 2011-2015

	Past month e-cigarette use, females, grades 6-12, % (SE)				
	**n**	**White**	**Black**	**Hispanic/Latina**	**Other[1]**
All years	50,034	4.6 (0.3)	2.6* (0.3)	5.3 (0.4)	3.5 (0.5)
2011	9,315	0.6 (0.1)	0.4 (0.2)	0.5 (0.2)	0.2 (0.1)
2012	12,275	1.7 (0.2)	0.4* (0.2)	1.6 (0.4)	0.9 (0.6)
2013	9,177	2.5 (0.4)	1.3 (0.4)	3.0 (0.5)	1.8 (0.7)
2014	10,645	8.8 (0.9)	4.1* (0.9)	9.8 (1.2)	5.8 (1.4)
2015	8,622	9.4 (1.0)	7.0 (1.1)	10.9 (1.0)	9.0 (2.1)
Trend[2]		<0.001	<0.001	<0.001	<0.001
	**Past month e-cigarette use, males, grades 6-12, % (SE)**				
	**n**	**White**	**Black**	**Hispanic/Latino**	**Other[1]**
All years	50,977	6.7 (0.3)	3.7* (0.3)	7.4 (0.4)	5.9 (1.1)
2011	9,284	1.9 (0.3)	0.8 (0.5)	1.5 (0.4)	1.1 (0.6)
2012	12,369	3.0 (0.4)	1.9 (0.6)	3.2 (0.6)	1.9 (0.8)
2013	9,216	3.8 (0.5)	2.9 (0.7)	4.2 (0.7)	3.1 (1.2)
2014	11,150	11.3 (1.1)	5.9* (1.0)	12.1 (1.3)	8.0 (1.6)
2015	8,958	14.0 (1.0)	7.1* (1.2)	14.4 (1.2)	16.3 (5.4)
Trend[2]		<0.001	<0.001	<0.001	<0.001

S1 Table shows the prevalence of past 30-day e-cigarette use in the National Youth Tobacco Survey according to categories of race/ethnicity. E-cigarette use increased in all race/ethnicity categories. Generally, the lowest prevalence of past 30-day use was among individuals who identified as Black.

1. Individuals in other category identified as Asian (75.2%), American Indian/Alaska Native (14.0%), and Native Hawaiian/Other Pacific Islander (10.8%).
2. P-for-linear trend, 2011-2015

*Statistically significant (P < 0.001) difference in prevalence of past 30-day e-cigarettes use versus individuals who identified as White in that survey year

Marginal percentages adjusted across years for grade in school. Standard errors account for sampling design and multiple imputation.
