# Supplementary material for: Trends in characteristics and multi-product use among adolescents who use electronic cigarettes, United States 2011-2015
Source: PLoS One. 2017 May 5;12(5):e0177073. doi: 10.1371/journal.pone.0177073 (PMC5419603; doi:10.1371/journal.pone.0177073)
Supplement: S2 File — (PDF) [file pone.0177073.s002.pdf]

Supplemental Tables: Sensitivity Analyses

S2. Table 1. Electronic cigarette ever use and past month use according to ever use (yes or no) and past month use (yes or no) of other tobacco products, 2011-2015

COMPLETE CASE ANALYSIS: participants with missing values excluded; no imputation

	**Females, grades 6-12**											
	Overall (n: sample size)		Ever use of any other tobacco[1]				p-value[2]	Past 30-day use of any other tobacco[1]				p-value[2]
			Yes (n: sample size)		No (n: sample size)			Yes (n: sample size)		No (n: sample size)		
Ever use of e-cigarettes:												
	n	% e-cigarette ever (SE)	n	% e-cigarette ever (SE)	n	% e-cigarette ever (SE)		n	% e-cigarette ever (SE)	n	% e-cigarette ever (SE)	
All years	44,010	7.9 (0.3)	12,335	24.3 (0.7)	31,675	1.3 (0.2)	<0.001	4,971	35.2 (1.0)	39,039	4.2 (0.2)	<0.001
2011	8,068	2.4 (0.3)	2,470	7.1 (0.7)	5,598	0.2 (0.1)	<0.001	1,005	12.6 (1.4)	7,063	0.8 (0.2)	<0.001
2012	11,063	5.5 (0.6)	3,070	17.6 (1.2)	7,993	0.5 (0.2)	<0.001	1,270	27.3 (2.1)	9,793	2.5 (0.3)	<0.001
2013	7,985	7.0 (0.6)	2,357	22.0 (1.9)	5,628	0.7 (0.2)	<0.001	951	33.8 (3.7)	7,034	3.2 (0.5)	<0.001
2014	9,279	17.8 (1.6)	2,449	50.8 (2.2)	6,830	4.3 (0.8)	<0.001	1,006	66.5 (3.2)	8,273	11.3 (1.3)	<0.001
2015	7,615	23.6 (0.9)	1,989	66.9 (1.6)	5,626	7.6 (0.5)	<0.001	739	81.7 (2.1)	6,876	17.4 (0.8)	<0.001
Trend[3]		<0.001		<0.001		<0.001			<0.001		<0.001	
Past month use of e-cigarettes:												
	n	% e-cig. past month (SE)	n	% e-cig. past month (SE)	n	% e-cig. past month (SE)		n	% e-cig. past month (SE)	n	% e-cig. past month (SE)	
All years	44,010	3.0 (0.2)	12,335	9.4 (0.5)	31,675	0.5 (0.1)	<0.001	4,971	17.9 (0.8)	39,039	1.0 (0.1)	<0.001
2011	8,068	0.6 (0.1)	2,470	1.6 (0.3)	5,598	0.1 (0.0)	<0.001	1.005	3.4 (0.7)	7,063	0.1 (0.1)	<0.001
2012	11,063	1.4 (0.2)	3,070	4.6 (0.5)	7,993	0.1 (0.1)	<0.001	1,270	10.5 (1.2)	9,793	0.2 (0.1)	<0.001
2013	7,985	2.4 (0.3)	2,357	7.5 (1.0)	5,628	0.3 (0.2)	<0.001	951	14.8 (2.1)	7,034	0.7 (0.2)	<0.001
2014	9,279	8.0 (0.9)	2,449	23.8 (2.0)	6,830	1.5 (0.4)	<0.001	1,006	43.2 (3.2)	8,273	3.3 (0.6)	<0.001
2015	7,615	8.6 (0.6)	1,989	27.3 (1.4)	5,626	1.6 (0.2)	<0.001	739	52.5 (3.0)	6,876	3.9 (0.4)	<0.001
Trend[3]		<0.001		<0.001		<0.001			<0.001		<0.001	
	**Males, grades 6-12**											
	Overall (n: sample size)		Ever use of any other tobacco[1]				p-value[2]	Past 30-day use of any other tobacco[1]				p-value[2]
			Yes (n: sample size)		No (n: sample size)			Yes (n: sample size)		No (n: sample size)		
Ever use of e-cigarettes:												
	n	% e-cigarette ever (SE)	n	% e-cigarette ever (SE)	n	% e-cigarette ever (SE)		n	% e-cigarette ever (SE)	n	% e-cigarette ever (SE)	
All years	42,232	10.2 (0.3)	14,026	26.7 (0.7)	28,206	1.6 (0.1)	<0.001	6,784	37.8 (1.0)	35,448	4.5 (0.2)	<0.001
2011	7,553	3.9 (0.5)	2,831	9.9 (1.1)	4,722	0.4 (0.2)	<0.001	1,385	17.3 (1.9)	6,168	0.9 (0.3)	<0.001
2012	10,532	7.7 (0.6)	3,596	21.8 (1.3)	6,936	0.4 (0.1)	<0.001	1,822	34.1 (2.0)	8,710	2.4 (0.5)	<0.001
2013	7,452	9.2 (0.6)	2,557	25.9 (1.3)	4,895	0.6 (0.2)	<0.001	1,227	41.4 (1.7)	6,225	3.2 (0.5)	<0.001
2014	9,269	20.1 (0.9)	2,861	52.8 (1.8)	6,408	5.2 (0.6)	<0.001	1,332	65.1 (2.5)	7,937	12.7 (1.2)	<0.001
2015	7,426	27.4 (1.0)	2,181	70.3 (1.4)	5,245	9.3 (0.6)	<0.001	1,018	79.9 (2.2)	6,408	18.8 (0.9)	<0.001
Trend[3]		<0.001		<0.001		<0.001			<0.001		<0.001	
Past month use of e-cigarettes:												
	n	% e-cig. past month (SE)	n	% e-cig. past month (SE)	n	% e-cig. past month (SE)		n	% e-cig. past month (SE)	n	% e-cig. past month (SE)	
All years	42,232	4.3 (0.2)	14,026	11.7 (0.6)	28,206	0.6 (0.1)	<0.001	6,784	19.6 (0.9)	35,448	1.3 (0.1)	<0.001
2011	7,553	1.5 (0.2)	2,831	3.9 (0.6)	4,722	0.2 (0.1)	<0.001	1,385	7.1 (1.1)	6,168	0.2 (0.1)	<0.001
2012	10,532	2.7 (0.3)	3,596	7.7 (0.8)	6,936	0.2 (0.1)	<0.001	1,822	13.8 (1.4)	8,710	0.4 (0.1)	<0.001
2013	7,452	3.7 (0.4)	2,557	10.3 (1.0)	4,895	0.3 (0.2)	<0.001	1,227	19.8 (1.8)	6,225	0.6 (0.2)	<0.001
2014	9,269	9.6 (0.8)	2,861	26.9 (2.0)	6,408	1.8 (0.3)	<0.001	1,332	42.8 (3.2)	7,937	4.1 (0.6)	<0.001
2015	7,426	11.8 (0.7)	2,181	33.5 (1.8)	5,245	2.7 (0.3)	<0.001	1,018	52.7 (3.1)	6,408	5.2 (0.5)	<0.001
Trend[3]		<0.001		<0.001		<0.001			<0.001		<0.001	

In all years, e-cigarette use (ever and past 30-days) was associated with use of other tobacco products, but e-cigarette use prevalence increased from 2011-2015 among both other tobacco users and other tobacco non-users.

1. Other tobacco includes: cigarettes, cigars, pipes, bidis, kreteks (2011-2013), conventional smokeless tobacco, snus, dissolvable tobacco, and hookah.
2. Chi-square test for difference in use of e-cigarettes between users and non-users of other tobacco products
3. P-for-linear trend, 2011-2015

Marginal percentages adjusted across years for grade in school and race/ethnicity.

Abbreviations: e-cig. = e-cigarette; n = sample size (denominator); SE = standard error

S2. Table 2. Past month use of other tobacco products according to past month use (yes or no) of electronic cigarettes, 2011-2015

COMPLETE CASE ANALYSIS: participants with missing values excluded; no imputation

	**Females, grades 6-12**							**Males, grades 6-12**						
	Overall (n: sample size)		Past 30-day e-cigarette use				1	Overall (n: sample size)		Past 30-day e-cigarette use				1
			Yes (n: sample size)		No (n: sample size)					Yes (n: sample size)		No (n: sample size)		
Any other tobacco in past month:														
	n	% other tobacco (SE)	n	% other tobacco (SE)	n	% other tobacco (SE)		n	% other tobacco (SE)	n	% other tobacco (SE)	n	% other tobacco (SE)	
All years	44,010	11.3 (0.3)	1,693	65.1 (1.9)	42,317	9.0 (0.2)	*	42,232	16.2 (0.4)	2,421	70.1 (1.2)	39,811	12.9 (0.4)	*
2011	8,068	12.6 (0.7)	56	79.3 (7.1)	8,012	12.2 (0.7)	*	7,553	18.9 (0.9)	97	88.2 (3.7)	7,456	17.6 (0.7)	*
2012	11,063	11.6 (0.6)	146	93.6 (2.4)	10,917	10.4 (0.6)	*	10,532	17.6 (0.8)	284	88.8 (2.6)	10,248	15.5 (0.5)	*
2013	7,985	11.6 (0.6)	180	75.2 (4.3)	7,805	10.0 (0.5)	*	7,452	16.4 (0.9)	262	87.7 (2.2)	7,190	13.6 (0.6)	*
2014	9,279	10.9 (0.5)	676	63.2 (2.8)	8,603	6.7 (0.5)	*	9,269	14.6 (0.7)	888	65.8 (2.2)	8,381	9.5 (0.5)	*
2015	7,615	9.6 (0.7)	635	58.4 (3.3)	6,980	5.1 (0.5)	*	7,426	13.8 (0.8)	890	61.4 (2.2)	6,536	7.6 (0.8)	*
Trend[2]		0.014		<0.001		<0.001			0.001		0.002		<0.001	
Cigarettes in past month:														
	n	% cigarettes (SE)	n	% cigarettes (SE)	n	% cigarettes (SE)		n	% cigarettes (SE)	n	% cigarettes (SE)	n	% cigarettes (SE)	
All years	44,010	6.8 (0.2)	1,693	41.9 (1.6)	42,317	5.3 (0.2)	*	42,232	8.6 (0.3)	2,421	43.3 (1.3)	39,811	6.4 (0.2)	*
2011	8,068	9.2 (0.7)	56	70.9 (7.6)	8,012	8.7 (0.7)	*	7,553	11.2 (0.7)	97	71.6 (5.5)	7,456	10.2 (0.7)	*
2012	11,063	7.4 (0.5)	146	84.3 (4.5)	10,917	6.3 (0.4)	*	10,532	10.0 (0.6)	284	76.6 (3.1)	10,248	8.1 (0.5)	*
2013	7,985	7.3 (0.5)	180	65.3 (4.6)	7,805	5.9 (0.4)	*	7,452	8.7 (0.6)	262	67.5 (4.0)	7,190	6.5 (0.6)	*
2014	9,279	5.1 (0.3)	676	37.5 (2.2)	8,603	2.4 (0.3)	*	9,269	6.6 (0.6)	888	36.5 (1.9)	8,381	3.6 (0.5)	*
2015	7,615	4.9 (0.5)	635	31.3 (2.9)	6,980	2.5 (0.4)	*	7,426	6.1 (0.6)	890	29.6 (2.4)	6,536	3.0 (0.5)	*
Trend[2]		<0.001		<0.001		<0.001			<0.001		<0.001		<0.001	
Non-cigarette combustibles[3] in past month:														
	n	% combustible (SE)	n	% combustible (SE)	n	% combustible (SE)		n	% combustible (SE)	n	% combustible (SE)	n	% combustible (SE)	
All years	44,010	5.4 (0.2)	1,693	29.5 (1.3)	42,317	4.4 (0.2)	*	42,232	9.3 (0.2)	2,421	43.1 (1.2)	39,811	7.2 (0.2)	*
2011	8,068	6.3 (0.4)	56	40.1 (8.6)	8,012	6.1 (0.4)	*	7,553	11.0 (0.5)	97	64.2 (6.2)	7,456	10.0 (0.5)	*
2012	11,063	6.6 (0.4)	146	54.9 (4.2)	10,917	5.9 (0.4)	*	10,532	11.1 (0.5)	284	69.1 (3.8)	10,248	9.4 (0.5)	*
2013	7,985	6.5 (0.4)	180	47.5 (4.9)	7,805	5.5 (0.4)	*	7,452	10.3 (0.6)	262	62.9 (3.0)	7,190	8.3 (0.5)	*
2014	9,279	3.9 (0.3)	676	26.2 (2.3)	8,603	2.0 (0.2)	*	9,269	7.0 (0.5)	888	37.4 (2.2)	8,381	3.9 (0.4)	*
2015	7,615	4.0 (0.3)	635	22.9 (1.9)	6,980	2.2 (0.3)	*	7,426	6.8 (0.5)	890	32.7 (1.8)	6,536	3.5 (0.4)	*
Trend[2]		<0.001		<0.001		<0.001			<0.001		<0.001		<0.001	
Smokeless tobacco[4] in past month:														
	n	% smokeless (SE)	n	% smokeless (SE)	n	% smokeless (SE)		n	% smokeless (SE)	n	% smokeless (SE)	n	% smokeless (SE)	
All years	44,010	1.5 (0.1)	1,693	12.2 (0.9)	42,317	1.0 (0.1)	*	42,232	6.8 (0.3)	2,421	31.3 (1.5)	39,811	5.3 (0.3)	*
2011	8,068	1.8 (0.2)	56	21.2 (7.2)	8,012	1.7 (0.2)		7,553	8.5 (0.8)	97	51.5 (6.7)	7,456	7.7 (0.8)	*
2012	11,063	1.8 (0.2)	146	28.5 (3.6)	10,917	1.4 (0.2)	*	10,532	7.4 (0.6)	284	46.6 (3.7)	10,248	6.3 (0.5)	*
2013	7,985	1.4 (0.2)	180	18.7 (3.8)	7,805	0.9 (0.2)	*	7,452	6.0 (0.7)	262	30.0 (3.9)	7,190	5.1 (0.7)	*
2014	9,279	1.2 (0.1)	676	9.8 (1.2)	8,603	0.5 (0.1)	*	9,269	6.4 (0.6)	888	31.0 (2.9)	8,381	3.9 (0.5)	*
2015	7,615	1.3 (0.3)	635	9.4 (1.4)	6,980	0.6 (0.2)	*	7,426	5.7 (0.7)	890	25.9 (2.5)	6,536	3.1 (0.6)	*
Trend[2]		0.058		<0.001		0.001			0.025		<0.001		<0.001	
Hookah in past month:														
	n	% hookah (SE)	n	% hookah (SE)	n	% hookah (SE)		n	% hookah (SE)	n	% hookah (SE)	n	% hookah (SE)	
All years	44,010	3.9 (0.1)	1,693	35.7 (2.1)	42,317	2.5 (0.1)	*	42,232	4.2 (0.2)	2,421	30.7 (1.5)	39,811	2.5 (0.1)	*
2011	8,068	2.4 (0.2)	56	17.5 (6.5)	8,012	2.3 (0.2)		7,553	3.0 (0.4)	97	32.7 (5.9)	7,456	2.5 (0.4)	*
2012	11,063	2.9 (0.3)	146	27.9 (3.7)	10,917	2.6 (0.3)	*	10,532	4.2 (0.3)	284	39.0 (3.5)	10,248	3.2 (0.3)	*
2013	7,985	3.2 (0.3)	180	29.6 (4.4)	7,805	2.5 (0.3)	*	7,452	3.5 (0.3)	262	31.6 (3.3)	7,190	2.4 (0.3)	*
2014	9,279	6.6 (0.4)	676	40.9 (3.5)	8,603	3.8 (0.4)	*	9,269	5.5 (0.4)	888	31.0 (2.9)	8,381	2.9 (0.3)	*
2015	7,615	4.4 (0.4)	635	34.9 (3.2)	6,980	1.6 (0.2)	*	7,426	4.6 (0.4)	890	27.9 (2.4)	6,536	1.6 (0.3)	*
Trend[2]		<0.001		0.098		0.906			<0.001		0.071		0.097	

1. Chi-square test for difference in other tobacco product use between past 30-day e-cigarette users and non-users (*P<0.001)
2. P-for-linear trend, 2011-2015
3. Non-cigarette combustibles include cigars, pipes, bidis, and kreteks (2011-2013)
4. Smokeless tobacco includes conventional smokeless tobacco, snus, and dissolvable tobacco

Marginal prevalences adjusted across years for grade in school and race/ethnicity

Abbreviations: n = sample size (denominator); SE = standard error

S2. Table 3. Cigarette use intensity and quit intentions according to past month use (yes or no) of electronic cigarettes, 2011-2015

COMPLETE CASE ANALYSIS: participants with missing values excluded; no imputation

	Females, grades 6-12							Males, grades 6-12						
	All past 30-day cigarette users (n: sample size)		Concurrent past 30-day e-cigarette use[1]					All past 30-day cigarette users (n: sample size)		Concurrent past 30-day e-cigarette use[1]				
			Yes (n: sample size)		No (n: sample size)					Yes (n: sample size)		No (n: sample size)		
Cigarettes all 30 days in past month:														
	n	% all 30 (SE)	n	% all 30 (SE)	n	% all 30 (SE)		n	% all 30 (SE)	n	% all 30 (SE)	n	% all 30 (SE)	
All years	2,932	19.9 (1.0)	727	26.4 (2.0)	2,205	17.7 (1.1)	*	3,635	22.7 (1.0)	1,077	29.5 (1.9)	2,558	19.8 (1.1)	*
2011	718	20.2 (2.3)	-[2]	-	679	19.3 (2.3)		844	25.2 (1.9)	70	54.5 (7.5)	774	22.0 (1.8)	
2012	814	18.8 (1.8)	124	31.2 (5.4)	690	16.6 (1.6)		1,054	22.8 (1.7)	221	40.1 (4.0)	833	18.4 (1.6)	*
2013	585	21.4 (2.4)	122	32.8 (4.7)	463	18.2 (2.6)		645	23.1 (2.1)	176	26.1 (3.9)	469	21.7 (2.7)	
2014	460	21.2 (2.5)	249	25.5 (3.7)	211	16.5 (3.1)		625	20.6 (2.6)	331	23.8 (3.9)	294	17.2 (3.3)	
2015	355	16.7 (2.4)	193	19.7 (3.4)	162	13.6 (3.0)		467	21.0 (3.2)	279	22.9 (3.0)	188	18.2 (6.2)	
Trend[3]		0.714		0.009		0.258			0.077		<0.001		0.118	
Smoked >10 cigarettes/day:														
	n	% 10/day (SE)	n	% 10/day (SE)	n	% 10/day (SE)		n	% 10/day (SE)	n	% 10/day (SE)	n	% 10/day (SE)	
All years	2,932	5.5 (0.5)	727	8.9 (1.2)	2,205	4.3 (0.5)	*	3,635	11.2 (0.7)	1,077	16.2 (1.6)	2,558	9.1 (0.7)	*
2011	718	5.1 (0.9)	-[2]	-	679	4.9 (0.9)		844	11.3 (1.2)	70	34.2 (7.5)	774	8.7 (1.0)	
2012	814	4.6 (0.8)	124	7.4 (2.8)	690	4.0 (0.8)		1,054	12.9 (1.4)	221	20.9 (3.3)	833	11.1 (1.4)	
2013	585	6.6 (1.3)	122	12.9 (3.9)	463	4.9 (1.2)		645	11.8 (1.3)	176	16.6 (3.4)	469	9.6 (1.6)	
2014	460	7.0 (1.4)	249	10.1 (2.2)	211	3.2 (1.3)		625	9.8 (2.3)	331	12.1 (3.6)	294	7.3 (2.8)	
2015	355	4.7 (1.2)	193	6.5 (2.4)	162	2.6 (1.4)		467	9.8 (1.6)	279	11.2 (2.0)	188	7.9 (3.1)	
Trend[3]		0.613		0.188		0.299			0.144		<0.001		0.261	
Cigarette quit attempt in past year:														
	n	% quit attempt (SE)	n	% quit attempt (SE)	n	% quit attempt (SE)		n	% quit attempt (SE)	n	% quit attempt (SE)	n	% quit attempt (SE)	
All years	2,932	59.4 (1.1)	727	61.7 (2.2)	2,205	58.6 (1.3)		3,635	56.5 (1.1)	1,077	55.8 (1.9)	2,558	56.8 (1.3)	
2011	718	58.6 (2.4)	-[2]	-	679	59.0 (2.5)		844	55.9 (2.4)	70	55.7 (6.7)	774	55.9 (2.4)	
2012	814	59.7 (2.2)	124	60.2 (6.0)	690	59.6 (2.2)		1,054	54.9 (1.9)	221	51.7 (4.6)	833	55.5 (2.3)	
2013	585	56.8 (2.2)	122	54.5 (4.8)	463	57.4 (2.6)		645	58.1 (2.3)	176	57.4 (4.1)	469	59.1 (2.9)	
2014	460	58.8 (2.7)	249	59.3 (3.7)	211	57.7 (4.5)		625	59.7 (2.9)	331	58.0 (4.2)	294	61.0 (4.3)	
2015	355	66.2 (4.0)	193	72.5 (5.3)	162	57.6 (5.5)		467	57.5 (4.0)	279	58.1 (5.3)	188	55.0 (5.4)	
Trend[3]		0.241		0.037		0.609			0.551		0.750		0.568	
Thinking about quitting cigarettes:														
	n	% think quit (SE)	n	% think quit (SE)	n	% think quit (SE)		n	% think quit (SE)	n	% think quit (SE)	n	% think quit (SE)	
All years	2,932	57.8 (1.3)	727	58.7 (2.4)	2,205	57.5 (1.6)		3,635	56.4 (1.2)	1,077	56.6 (2.0)	2,558	56.4 (1.4)	
2011	718	51.8 (3.3)	-[2]	-	679	52.0 (3.4)		844	45.5 (2.4)	70	43.1 (9.0)	774	45.7 (2.4)	
2012	814	64.0 (1.9)	124	63.0 (5.8)	690	64.3 (2.0)		1,054	64.6 (1.7)	221	55.2 (4.1)	833	67.4 (2.0)	
2013	585	55.4 (3.0)	122	50.6 (6.0)	463	56.8 (3.3)		645	56.0 (2.8)	176	57.3 (5.3)	469	56.1 (3.4)	
2014	460	60.0 (2.5)	249	59.0 (3.8)	211	60.4 (3.5)		625	61.8 (2.8)	331	59.2 (3.5)	294	63.9 (5.1)	
2015	355	61.5 (3.7)	193	62.8 (4.8)	162	59.9 (4.6)		467	61.0 (4.5)	279	61.6 (5.1)	188	59.5 (5.9)	
Trend[3]		0.108		0.459		0.173			0.006		0.170		0.006	
Thinking about quitting all tobacco:														
	n	% think quit (SE)	n	% think quit (SE)	n	% think quit (SE)		n	% think quit (SE)	n	% think quit (SE)	n	% think quit (SE)	
All years	2,932	52.6 (1.3)	727	46.6 (2.2)	2,205	54.9 (1.5)		3,635	46.9 (1.1)	1,077	43.0 (2.1)	2,558	48.7 (1.3)	
2011	718	54.8 (2.8)	-[2]	-	679	54.7 (2.8)		844	43.4 (2.0)	70	43.4 (7.7)	774	43.4 (2.0)	
2012	814	59.2 (2.3)	124	53.0 (5.7)	690	60.2 (2.4)		1,054	55.8 (2.0)	221	50.7 (4.5)	833	57.2 (2.2)	
2013	585	50.4 (2.9)	122	47.0 (5.3)	463	51.7 (3.3)		645	50.4 (3.0)	176	48.2 (5.5)	469	52.0 (3.2)	
2014	460	42.8 (2.6)	249	38.7 (4.0)	211	48.8 (4.1)		625	41.5 (3.0)	331	39.0 (3.4)	294	43.7 (4.9)	
2015	355	54.6 (4.7)	193	52.0 (4.1)	162	57.0 (6.5)		467	42.6 (4.1)	279	41.3 (5.6)	188	44.2 (5.0)	
Trend[3]		0.030		0.300		0.378			0.073		0.045		0.973	

Among past 30-day cigarette users, past 30-day e-cigarette use was not associated with quit attempts or intentions.

1. Chi-square test for difference in cigarette smoking intensity or quit behavior/intentions between past 30-day cigarette users with or without past 30-day e-cigarette use (**P*<0.001)
2. Estimates suppressed due to sample size (n < 50)
3. P-for-linear trend, 2011-2015

Marginal percentages adjusted across years for grade in school and race/ethnicity

Abbreviations: n = sample size (denominator); SE = standard error

S2. Table 4. Electronic cigarette ever use and past month use according to ever use (yes or no) and past month use (yes or no) of other tobacco products, 2011-2013

YEAR 2014 & 2015 DATA EXCLUDED

	Females, grades 6-12											
	Overall (n: sample size)		Ever use of any other tobacco[1]					Past 30-day use of any other tobacco[1]				
			Yes (n: sample size)		No (n: sample size)		p-value[2]	Yes (n: sample size)		No (n: sample size)		p-value[2]
Ever use of e-cigarettes:												
	n	% e-cigarette ever (SE)	n	% e-cigarette ever (SE)	n	% e-cigarette ever (SE)		n	% e-cigarette ever (SE)	n	% e-cigarette ever (SE)	
All years	30,767	5.0 (0.3)	9,644	15.2 (0.6)	21,123	0.5 (0.1)	<0.001	4,003	23.9 (1.1)	26,764	2.2 (0.2)	<0.001
2011	9,315	2.5 (0.3)	3,091	7.2 (0.8)	6,224	0.2 (0.1)	<0.001	1,263	12.6 (1.4)	8,052	0.9 (0.2)	<0.001
2012	12,275	5.6 (0.5)	3,622	17.5 (1.1)	8,653	0.5 (0.1)	<0.001	1,510	27.0 (1.9)	10,765	2.5 (0.3)	<0.001
2013	9,177	7.0 (0.5)	2,931	21.7 (1.4)	6,246	0.7 (0.2)	<0.001	1,230	33.0 (2.4)	7,947	3.3 (0.4)	<0.001
Trend[3]		<0.001		<0.001		0.009			<0.001		<0.001	
Past month use of e-cigarettes:												
	n	% e-cig. past month (SE)	n	% e-cig. past month (SE)	n	% e-cig. past month (SE)		n	% e-cig. past month (SE)	n	% e-cig. past month (SE)	
All years	30,767	1.5 (0.1)	9,644	4.4 (0.3)	21,123	0.2 (0.1)	<0.001	4,003	9.2 (0.7)	26,764	0.3 (0.1)	<0.001
2011	9,315	0.6 (0.1)	3,091	1.6 (0.3)	6,224	0.1 (0.0)	<0.001	1,263	3.4 (0.7)	8,052	0.1 (0.1)	<0.001
2012	12,275	1.4 (0.2)	3,622	4.5 (0.5)	8,653	0.1 (0.1)	<0.001	1,510	10.1 (1.1)	10,765	0.2 (0.1)	<0.001
2013	9,177	2.4 (0.3)	2,931	7.3 (0.8)	6,246	0.3 (0.2)	<0.001	1,230	14.4 (1.8)	7,947	0.7 (0.1)	<0.001
Trend[3]		<0.001		<0.001		0.146			<0.001		<0.001	
	**Males, grades 6-12**											
	Overall (n: sample size)		Ever use of any other tobacco[1]					Past 30-day use of any other tobacco[1]				
			Yes (n: sample size)		No (n: sample size)		p-value[2]	Yes (n: sample size)		No (n: sample size)		p-value[2]
Ever use of e-cigarettes:												
	n	% e-cigarette ever (SE)	n	% e-cigarette ever (SE)	n	% e-cigarette ever (SE)		n	% e-cigarette ever (SE)	n	% e-cigarette ever (SE)	
All years	30,869	7.4 (0.3)	11,797	18.7 (0.7)	19,072	0.5 (0.1)	<0.001	5,950	28.5 (1.1)	24,919	2.3 (0.2)	<0.001
2011	9,284	4.2 (0.5)	3,793	10.1 (1.2)	5,491	0.5 (0.2)	<0.001	1,897	16.7 (1.8)	7,387	1.0 (0.2)	<0.001
2012	12,369	8.3 (0.6)	4,566	21.7 (1.3)	7,803	0.4 (0.1)	<0.001	2,363	32.5 (2.1)	10,006	2.5 (0.3)	<0.001
2013	9,216	9.6 (0.6)	3,438	24.6 (1.4)	5,778	0.7 (0.2)	<0.001	1,690	37.6 (1.9)	7,526	3.3 (0.4)	<0.001
Trend[3]		<0.001		<0.001		0.394			<0.001		<0.001	
Past month use of e-cigarettes:												
	n	% e-cig. past month (SE)	n	% e-cig. past month (SE)	n	% e-cig. past month (SE)		n	% e-cig. past month (SE)	n	% e-cig. past month (SE)	
All years	30,869	2.7 (0.2)	11,797	6.9 (0.5)	19,072	0.2 (0.1)	<0.001	5,950	12.3 (0.9)	24,919	0.4 (0.1)	<0.001
2011	9,284	1.6 (0.3)	3,793	3.8 (0.6)	5,491	0.2 (0.1)	<0.001	1,897	6.9 (1.1)	7,387	0.2 (0.1)	<0.001
2012	12,369	2.8 (0.4)	4,566	7.3 (0.8)	7,803	0.2 (0.1)	<0.001	2,363	12.8 (1.4)	10,006	0.4 (0.1)	<0.001
2013	9,216	3.7 (0.4)	3,438	9.6 (1.0)	5,778	0.3 (0.1)	<0.001	1,690	17.8 (1.8)	7,526	0.7 (0.1)	<0.001
Trend[3]		<0.001		<0.001		0.355			<0.001		0.013	

In all years, e-cigarette use was associated with use of other tobacco products, but e-cigarette use prevalence increased among both other tobacco users and other tobacco non-users.

1. Other tobacco includes: cigarettes, cigars, pipes, bidis, kreteks (2011-2013), conventional smokeless tobacco, snus, dissolvable tobacco, and hookah.

2. Chi-square test for difference in use of e-cigarettes between users and non-users of other tobacco products

3. P-for-linear trend, 2011-2013

Marginal percentages adjusted across years for grade in school and race/ethnicity.

Subgroup sample sizes (n) are the mean sample size over 10 imputations for missing value assignment.

Abbreviations: n = sample size (denominator); SE = standard error

S2. Table 5. Past month use of other tobacco products according to past month use (yes or no) of electronic cigarettes, 2011-2013

YEAR 2014 & 2015 DATA EXCLUDED

	**Females, grades 6-12**							**Males, grades 6-12**						
	Overall (n: sample size)		Past 30-day e-cigarette use				[1]	Overall (n: sample size)		Past 30-day e-cigarette use				[1]
			Yes (n: sample size)		No (n: sample size)					Yes (n: sample size)		No (n: sample size)		
Any other tobacco in past month:														
	n	% other tobacco (SE)	n	% other tobacco (SE)	n	% other tobacco (SE)		n	% other tobacco (SE)	n	% other tobacco (SE)	n	% other tobacco (SE)	
All years	30,767	12.9 (0.4)	462	80.6 (3.0)	30,305	11.9 (0.4)	*	30,869	19.3 (0.5)	804	86.8 (1.8)	30,065	17.4 (0.5)	*
2011	9,315	13.4 (0.8)	69	81.8 (7.4)	9,246	13.0 (0.7)	*	9,284	20.6 (1.0)	130	88.7 (4.2)	9,154	19.4 (1.0)	*
2012	12,275	12.6 (0.7)	180	89.4 (2.9)	12,095	11.4 (0.6)	*	12,369	19.3 (0.8)	342	88.0 (3.0)	12,027	17.3 (0.8)	*
2013	9,177	12.8 (0.6)	213	74.5 (5.0)	8,964	11.3 (0.6)	*	9,216	18.0 (0.9)	332	85.0 (2.7)	8,884	15.4 (1.0)	*
Trend[2]		0.515		0.212		0.067			0.061		0.398		0.004	
Cigarettes in past month:														
	n	% cigarettes (SE)	n	% cigarettes (SE)	n	% cigarettes (SE)		n	% cigarettes (SE)	n	% cigarettes (SE)	n	% cigarettes (SE)	
All years	30,767	8.6 (0.4)	462	70.9 (3.2)	30,305	7.6 (0.3)	*	30,869	10.9 (0.4)	804	70.5 (2.7)	30,065	9.3 (0.4)	*
2011	9,315	9.7 (0.8)	69	75.4 (7.8)	9,246	9.3 (0.8)	*	9,284	12.2 (0.8)	130	73.5 (6.2)	9,154	11.1 (0.8)	*
2012	12,275	8.1 (0.6)	180	79.2 (4.7)	12,095	7.1 (0.5)	*	12,369	11.1 (0.7)	342	74.9 (3.8)	12,027	9.2 (0.6)	*
2013	9,177	7.8 (0.5)	213	64.7 (5.2)	8,964	6.4 (0.5)	*	9,216	9.6 (0.7)	332	65.9 (4.5)	8,884	7.4 (0.6)	*
Trend[2]		0.056		0.167		0.002			0.012		0.229		<0.001	
Non-cigarette combustibles[3] in past month:														
	n	% combustible (SE)	n	% combustible (SE)	n	% combustible (SE)		n	% combustible (SE)	n	% combustible (SE)	n	% combustible (SE)	
All years	30,767	7.2 (0.1)	462	48.8 (4.1)	30,305	6.6 (0.3)	*	30,869	12.1 (0.3)	804	64.9 (2.4)	30,065	10.6 (0.3)	*
2011	9,315	6.9 (0.4)	69	44.7 (10.1)	9,246	6.7 (0.4)	*	9,284	12.5 (0.6)	130	66.4 (6.3)	9,154	11.5 (0.5)	*
2012	12,275	7.3 (0.5)	180	53.1 (4.9)	12,095	6.6 (0.4)	*	12,369	12.3 (0.6)	342	68.0 (4.1)	12,027	10.6 (0.5)	*
2013	9,177	7.5 (0.5)	213	47.1 (6.1)	8,964	6.5 (0.5)	*	9,216	11.6 (0.6)	332	61.9 (3.4)	8,884	9.7 (0.6)	*
Trend[2]		0.387		0.894		0.783			0.336		0.388		0.027	
Smokeless tobacco[4] in past month:														
	n	% smokeless (SE)	n	% smokeless (SE)	n	% smokeless (SE)		n	% smokeless (SE)	n	% smokeless (SE)	n	% smokeless (SE)	
All years	30,767	2.0 (0.1)	462	23.6 (3.5)	30,305	1.7 (0.1)	*	30,869	8.0 (0.4)	804	38.9 (2.7)	30,065	7.2 (0.4)	*
2011	9,315	2.1 (0.2)	69	27.1 (8.9)	9,246	2.0 (0.2)		9,284	9.2 (0.9)	130	52.0 (7.2)	9,154	8.4 (0.9)	*
2012	12,275	2.2 (0.2)	180	28.9 (4.5)	12,095	1.8 (0.2)	*	12,369	8.2 (0.6)	342	45.2 (3.9)	12,027	7.1 (0.6)	*
2013	9,177	1.8 (0.2)	213	19.6 (5.2)	8,964	1.3 (0.2)	*	9,216	6.7 (0.7)	332	28.8 (3.8)	8,884	5.9 (0.7)	*
Trend[2]		0.264		0.287		0.035			0.046		0.002		0.027	
Hookah in past month:														
	n	% hookah (SE)	n	% hookah (SE)	n	% hookah (SE)		n	% hookah (SE)	n	% hookah (SE)	n	% hookah (SE)	
All years	30,767	2.9 (0.2)	462	27.6 (3.0)	30,305	2.6 (0.2)	*	30,869	3.7 (0.2)	804	32.9 (2.5)	30,065	2.9 (0.2)	*
2011	9,315	2.5 (0.3)	69	20.7 (7.7)	9,246	2.3 (0.3)		9,284	3.2 (0.4)	130	31.9 (6.0)	9,154	2.7 (0.4)	*
2012	12,275	3.0 (0.3)	180	27.9 (4.2)	12,095	2.7 (0.3)	*	12,369	4.2 (0.4)	342	37.6 (4.1)	12,027	3.3 (0.3)	*
2013	9,177	3.3 (0.3)	213	29.0 (4.8)	8,964	2.7 (0.3)	*	9,216	3.6 (0.3)	332	29.8 (3.5)	8,884	2.6 (0.3)	*
Trend[2]		0.038		0.422		0.347			0.428		0.540		0.821	

As past month e-cigarette use increased, use became less exclusively associated with combustible tobacco use.

1. Chi-square test for difference in use of other tobacco products between past 30-day e-cigarette users and non-users (*$P<0.001$)

2. P-for-linear trend, 2011-2013

3. Non-cigarette combustibles include cigars, pipes, bidis, and kreteks (2011-2013)

4. Smokeless tobacco includes conventional smokeless tobacco, snus, and dissolvable tobacco

Marginal prevalences adjusted across years for grade in school and race/ethnicity

Subgroup sample sizes (n) are the mean sample size over 10 imputations for missing value assignment.

Abbreviations: n = sample size (denominator); SE = standard error

S2. Table 6. Cigarette use intensity and quit intentions according to past month use (yes or no) of electronic cigarettes, 2011-2013

YEAR 2014 & 2015 DATA EXCLUDED

	Females, grades 6-12							Males, grades 6-12						
	All past 30-day cigarette users (n: sample size)		Concurrent past 30-day e-cigarette use [1]					All past 30-day cigarette users (n: sample size)		Concurrent past 30-day e-cigarette use [1]				
			Yes (n: sample size)		No (n: sample size)					Yes (n: sample size)		No (n: sample size)		
Cigarettes all 30 days in past month:														
	n	% all 30 (SE)	n	% all 30 (SE)	n	% all 30 (SE)		n	% all 30 (SE)	n	% all 30 (SE)	n	% all 30 (SE)	
All years	3,180	21.3 (1.1)	642	34.0 (4.1)	2,538	19.4 (1.3)	*	4,280	25.2 (1.1)	1,053	36.9 (3.0)	3,227	22.6 (1.2)	*
2011	893	21.1 (1.9)	-[2]	-	847	20.1 (2.0)		1,165	26.4 (1.8)	93	53.3 (7.5)	1,072	23.3 (2.0)	*
2012	970	20.2 (1.8)	145	31.1 (6.6)	825	18.3 (2.0)		1,349	23.8 (1.8)	260	38.6 (4.7)	1,089	20.2 (1.9)	*
2013	740	22.6 (2.3)	143	34.7 (5.6)	597	19.5 (2.9)		894	25.3 (2.1)	230	27.6 (4.6)	664	24.5 (2.8)	
Trend[3]		0.658		0.931		0.814			0.644		0.004		0.849	
Smoked >10 cigarettes/day:														
	n	% 10/day (SE)	n	% 10/day (SE)	n	% 10/day (SE)		n	% 10/day (SE)	n	% 10/day (SE)	n	% 10/day (SE)	
All years	3,180	6.8 (0.6)	642	12.6 (3.4)	2,538	5.9 (0.8)		4,280	13.8 (0.7)	1,053	22.8 (2.7)	3,227	11.8 (0.8)	*
2011	893	6.1 (0.8)	-[2]	-	847	5.7 (0.9)		1,165	13.6 (1.3)	93	37.2 (8.1)	1,072	10.7 (1.4)	*
2012	970	6.4 (1.0)	145	8.7 (4.6)	825	5.9 (1.3)		1,349	13.9 (1.3)	260	20.6 (3.9)	1,089	12.5 (1.5)	
2013	740	8.2 (1.3)	143	16.1 (5.4)	597	6.1 (1.8)		894	14.0 (1.4)	230	17.6 (3.8)	664	12.6 (1.8)	
Trend[3]		0.195		0.314		0.846			0.837		0.040		0.394	
Cigarette quit attempt in past year:														
	n	% quit attempt (SE)	n	% quit attempt (SE)	n	% quit attempt (SE)		n	% quit attempt (SE)	n	% quit attempt (SE)	n	% quit attempt (SE)	
All years	3,180	59.8 (1.4)	642	57.8 (3.8)	2,538	60.1 (1.5)		4,280	57.2 (1.2)	1,053	54.6 (3.0)	3,227	57.8 (1.4)	
2011	893	59.6 (2.4)	-[2]	-	847	60.0 (2.6)		1,165	57.6 (2.3)	93	57.1 (6.7)	1,072	57.6 (2.5)	
2012	970	61.9 (2.4)	145	60.6 (6.1)	825	61.9 (2.4)		1,349	55.6 (2.0)	260	51.9 (5.3)	1,089	56.5 (2.4)	
2013	740	58.0 (2.7)	143	56.7 (5.5)	597	58.2 (3.0)		894	58.6 (2.3)	230	55.7 (4.5)	664	59.9 (3.0)	
Trend[3]		0.718		0.966		0.737			0.812		0.987		0.609	
Thinking about quitting cigarettes:														
	n	% think quit (SE)	n	% think quit (SE)	n	% think quit (SE)		n	% think quit (SE)	n	% think quit (SE)	n	% think quit (SE)	
All years	3,180	57.3 (1.7)	642	55.1 (4.1)	2,538	57.6 (1.8)		4,280	56.4 (1.3)	1,053	54.9 (3.2)	3,227	56.8 (1.6)	
2011	893	52.6 (3.3)	-[2]	-	847	52.8 (3.3)		1,165	47.8 (2.5)	93	48.7 (8.8)	1,072	47.7 (2.6)	
2012	970	64.7 (1.9)	145	62.6 (6.0)	825	65.0 (2.1)		1,349	64.7 (1.8)	260	56.5 (5.3)	1,089	67.0 (2.2)	
2013	740	55.2 (3.2)	143	49.4 (6.5)	597	56.5 (3.5)		894	58.0 (2.6)	230	56.8 (4.9)	664	58.6 (3.2)	
Trend[3]		0.456		0.339		0.292			0.002		0.480		0.002	
Thinking about quitting all tobacco:														
	n	% think quit (SE)	n	% think quit (SE)	n	% think quit (SE)		n	% think quit (SE)	n	% think quit (SE)	n	% think quit (SE)	
All years	3,180	55.6 (1.6)	642	51.6 (4.1)	2,538	56.3 (1.8)		4,280	50.3 (1.3)	1,053	50.5 (3.5)	3,227	50.5 (1.4)	
2011	893	54.7 (2.8)	-[2]	-	847	54.6 (2.8)		1,165	46.3 (2.0)	93	49.9 (7.6)	1,072	46.0 (2.2)	
2012	970	60.2 (2.4)	145	54.3 (6.3)	825	61.2 (2.6)		1,349	54.2 (2.0)	260	51.7 (5.0)	1,089	55.2 (2.2)	
2013	740	52.1 (3.0)	143	48.0 (6.3)	597	53.5 (3.7)		894	50.9 (2.7)	230	49.9 (5.4)	664	51.8 (3.2)	
Trend[3]		0.615		0.391		0.978			0.132		0.948		0.078	

Among past 30-day cigarette users, past 30-day e-cigarette use was not associated with increased cigarette quit attempts or cessation intentions.

1. Chi-square test for difference in cigarette smoking intensity or quit behavior/intentions between past 30-day cigarette users with or without past 30-day e-cigarette use (*P<0.001)

2. Estimates suppressed due to sample size (n < 50)

3. P-for-linear trend, 2011-2013

Marginal percentages adjusted across years for grade in school and race/ethnicity

Subgroup sample sizes (n) are the mean sample size over 10 imputations for missing value assignment.

Abbreviations: n = sample size (denominator); SE = standard error
